# The master regulator of IncA/C plasmids is recognized by the *Salmonella* Genomic island SGI1 as a signal for excision and conjugal transfer

**DOI:** 10.1093/nar/gkv758

**Published:** 2015-10-10

**Authors:** János Kiss, Péter Pál Papp, Mónika Szabó, Tibor Farkas, Gábor Murányi, Erik Szakállas, Ferenc Olasz

**Affiliations:** Agricultural Biotechnology Institute, National Agricultural Research and Innovation Centre, Gödöllő H2100, Hungary

## Abstract

The genomic island SGI1 and its variants, the important vehicles of multi-resistance in *Salmonella* strains, are integrative elements mobilized exclusively by the conjugative IncA/C plasmids. Integration and excision of the island are carried out by the SGI1-encoded site-specific recombinase Int and the recombination directionality factor Xis. Chromosomal integration ensures the stable maintenance and vertical transmission of SGI1, while excision is the initial step of horizontal transfer, followed by conjugation and integration into the recipient. We report here that SGI1 not only exploits the conjugal apparatus of the IncA/C plasmids but also utilizes the regulatory mechanisms of the conjugation system for the exact timing and activation of excision to ensure efficient horizontal transfer. This study demonstrates that the FlhDC-family activator AcaCD, which regulates the conjugation machinery of the IncA/C plasmids, serves as a signal of helper entry through binding to SGI1 *xis* promoter and activating SGI1 excision. Promoters of *int* and *xis* genes have been identified and the binding site of the activator has been located by footprinting and deletion analyses. We prove that expression of *xis* is activator-dependent while *int* is constitutively expressed, and this regulatory mechanism is presumably responsible for the efficient transfer and stable maintenance of SGI1.

## INTRODUCTION

Outstanding plasticity of bacterial genomes enables the rapid adaptation to environmental changes. This flexibility is based to a great extent on the horizontal gene transfer (HGT) mechanisms by which bacteria can acquire and disseminate many beneficial traits such as unconventional metabolic pathways, virulence factors, resistance to antibiotics (AR) and heavy metals. One of the most efficient mechanisms in HGT is conjugation, which is widespread among naturally occurring plasmids, genomic islands (GI) and large transposons. Mobile GIs are classified into major groups of integrative conjugative elements (ICEs) and integrative mobilizable elements (IMEs; also known as mobilizable genomic islands, MGIs) according to their ability for self-transmission. Conjugation in Gram-negative bacteria requires the assembly of a type IV secretion system that establishes close cell-to-cell contact between the donor and recipient and a relaxosome complex that initiates DNA processing at the start site of conjugation (*oriT*) and transfers DNA to the secretion system. Conjugative plasmids and ICEs possess the complete genetic apparatus for encoding the components of these complexes, while IMEs and several mobilizable plasmids have *oriT* and a limited set of transfer genes, which is not sufficient for self-transmission, but enables hijacking the conjugation system of other transfer competent elements (1,2). Unlike plasmids, ICEs and IMEs can not maintain extrachromosomally, thus they must integrate into the host's chromosome by site-specific-, transposition- or homologous recombination to ensure their stable maintenance and vertical transmission.

**Figure 1.**
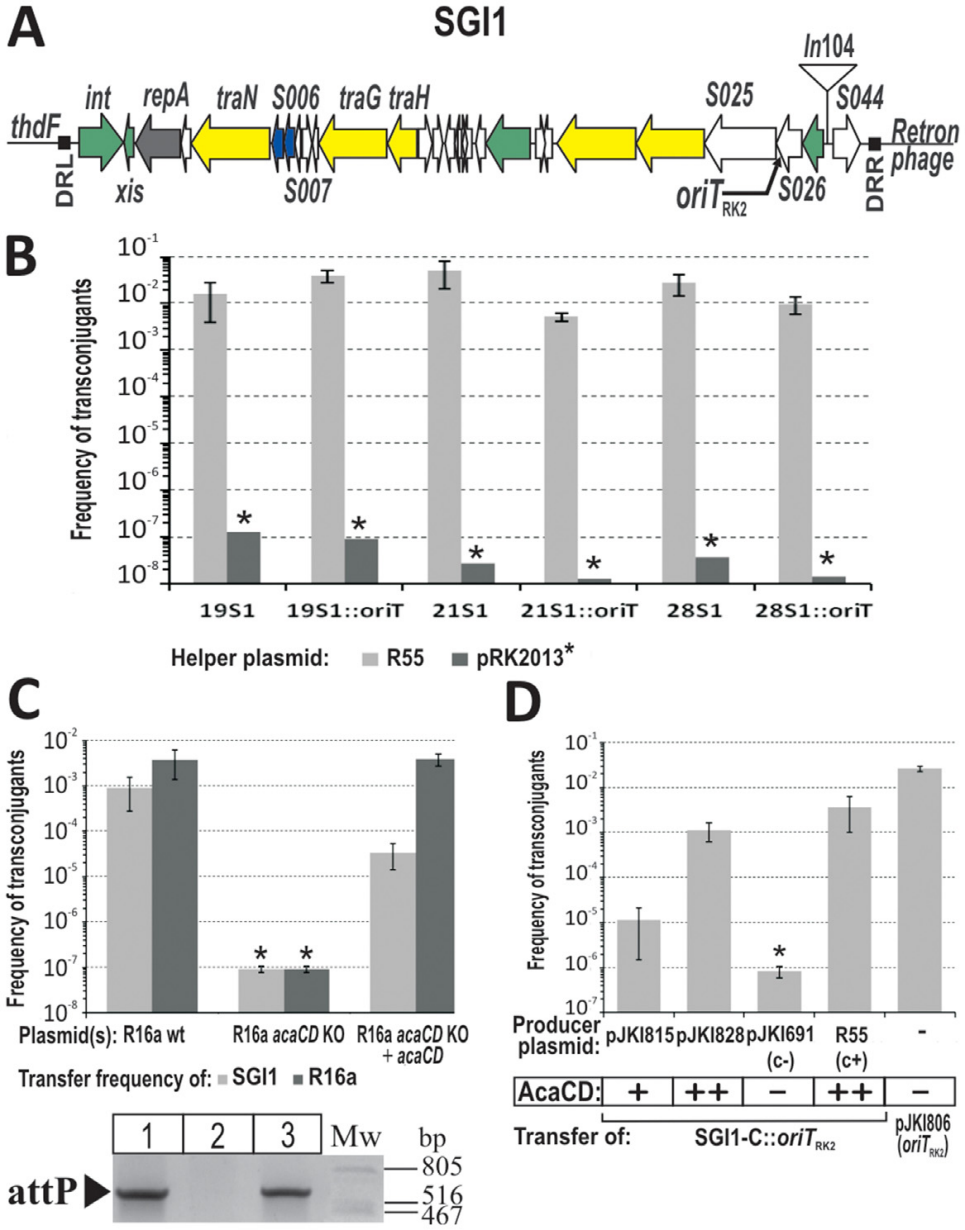
The role of *acaCD* in excision and conjugation of SGI1. (**A**) Schematic representation of SGI1 integrated at 3′ end of *thdF* in *S*. Typhimurium DT104. Direct repeats are shown as black boxes. Orfs with similar functions are colour coded: green, DNA recombination; grey, replication; yellow, conjugation; blue, regulator; white, unknown function. In104 region and the site of *oriT*_RK2_ insertion (see panels B and D) are indicated. (**B**) Mobilization of SGI1-C::*oriT*_RK2_ by RK2 transfer system. Conjugation frequency of wt SGI1-C and its derivative containing *oriT*_RK2_ was measured in three *S*. Typhimurium strains in the presence of R55 or pRK2013 helper plasmids, respectively. * SGI1 transfer frequency with pRK2013 helper plasmid was under the detection limit in each case. (**C**) The impact of *acaCD* deletion on the conjugation of helper plasmid R16a and SGI1-C. The conjugation of R16a *acaCD* KO plasmid (Text S4) and SGI1-C into *E. coli* TG2 recipient was measured with or without complementation. AcaCD was expressed from plasmid pJKI839. The gel image shows the attP specific (LJ2 – RJ4) PCR results of the donor strains in conjugation assay. Lane 1: TG1Nal::SGI1-C/R16a, lane 2: TG1Nal::SGI1-C/R16a *acaCD* KO, lane 3: TG1Nal::SGI1-C/R16a *acaCD* KO+pJKI839. (**D**) Complementation of the RK2 transfer system by AcaCD. Conjugation of SGI1-C::*oriT*_RK2_ was measured from *E. coli* donor strain S17–1Nal, which contains the whole transfer apparatus of RK2 integrated onto the chromosome, into TG2 recipient. AcaCD was expressed from plasmids pJKI815 or pJKI828. Plasmids pJKI691 and R55 were used as negative and positive controls, respectively. Control plasmid pJKI806 was applied to determine the transfer efficiency of *oriT*_RK2_ if it is located on a medium copy-number plasmid. *Conjugation frequency was under the detection limit. The relative expression levels of the activator are indicated.

Besides resistance plasmids, GIs are the major factors in rapid acquisition of multidrug resistance (MDR) phenotype by pathogenic bacteria such as *Vibrio* or *Salmonella* (3,4). *Salmonella* is one of the most prevalent zoonotic pathogens worldwide. The majority of human infections are caused by a few serotypes, such as *S. enterica* serovars Typhimurium or Enteritidis. A multiresistant clone of *S*. Typhimurium DT104 has become widespread since the early 1990s among humans and livestock (5) causing significant public health threats. The region responsible for the MDR phenotype of *S*. Typhimurium DT104 is located on the chromosomal Salmonella genomic island 1 (SGI1). This element was described as a 42.4-kb IME (6,4) containing 44 predicted orfs. Fifteen of these orfs, including genes associated with ampicillin, chloramphenicol/florfenicol, streptomycin/spectinomycin, sulphonamide and tetracycline (ACSSuT) resistant phenotype, reside in a complex class 1 integron segment In104 (Figure 1A). Since the identification of SGI1 prototype numerous variants that differ only in their MDR region (7) and cover a wide spectrum of antimicrobial resistance (8) have been described from several *S. enterica* serotypes and recently from *Proteus mirabilis* strains.

The site of SGI1 insertion (attB) is located in the 3′ end of *thdF* gene, which is followed by the *int2* gene of a retron phage in the *S*. Typhimurium chromosome or *yidY* in other SGI1^+^ serovars. The integrated island is flanked by imperfect 18-bp direct repeats DRL and DRR (9). DRR is identical to the last 18 bp of *thdF*, while DRL probably derives from the joined ends (attP) of the free circular form of SGI1 (10). The base changes in the end of *thdF* gene generated by SGI1 integration do not cause sequence alterations in the expressed protein. The λ integrase family member Int and the recombination directionality factor Xis, which are encoded near the 5′ end of SGI1, catalyse excision and integration of the island (Supplementary Figure S1A) (10). Although *int* and *xis* expression was reported (11), spontaneous excision was hardly detectable and SGI1 loss was not observed (6,7,9,12). Even though its backbone encodes several conjugation-related genes, SGI1 is not self-transmissible and only mobilized by conjugative plasmids belonging to the IncA/C incompatibility group (2,10). After conjugal transfer SGI1 integrates at the chromosomal attB site of the recipient *Salmonella* or *E. coli* or a secondary insertion site if attB is missing from the recipient strain (13). High frequency of SGI1 transfer was detected in the presence of IncA/C plasmids R55, R16a, IP40a or pVCR94 (2,7,14). Although the SGI1 transfer rate, ranging 10^−1^–10^−3^ per donor cfu, was comparable to that of the helper plasmids, their co-acquisition was much less frequent than expected (14).

The broad-host-range plasmids of IncA/C family are the most prevalent MDR carrying vectors among enteric bacteria, including potential zoonotic foodborne pathogens (such as *Salmonella, Klebsiella, Escherichia*) (15,16). Their rapid spread probably based on their efficient conjugative system and wide spectrum of resistance genes they deliver. These traits and the fact that they can also mobilize several MDR GIs represent a growing threat for human and animal healthcare. Comparative studies of IncA/C plasmids (16–19) showed that they share a >99% conserved backbone consisting of replication, maintenance, stability and transfer systems. Variability of the group derives from the accessory modules that are often large compound transposons harbouring various virulence factors, resistance genes embedded in integrons, or apparatus for detoxification of heavy metal compounds (20). The regulatory mechanisms controlling the conjugal transfer system of IncA/C plasmids have been recently characterized (14). The key player in this conserved regulatory cascade is the *flh*DC-like transcriptional activator called AcaCD, which is essential for the expression of transfer genes. Common motif has been determined in the promoter region of regulated genes and shown to be the core binding site of AcaCD. Similar motifs have been predicted in SGI1 and MGI*Vmi*1, a seemingly unrelated GI that is also mobilizable by IncA/C plasmids, and AcaCD has been shown to be involved in excision of these islands.

FlhDC-family regulators were primarily identified as master activators of flagellar operons in bacteria, however they have been adapted by several conjugative plasmids and ICEs to control their transfer apparatus. The regulatory mechanisms of the flagellar systems were the subject of extensive studies in *E. coli, Salmonella* and several other Gram-negative bacteria (21,22). This network of operons consists of more than sixty genes, which are regulated and expressed in a coordinated fashion and organized into a transcriptional hierarchy of three promoter classes. At the apex of this hierarchy is the operon containing master regulator genes *flh*D and *flh*C. Signals from different cellular networks (H-NS, OmpR, CRP, UmoABCD, Lrp) reflecting the metabolic state of the cell regulate the *flh*DC expression either negatively or positively (23,24). The gene products FlhD and FlhC appear to form a heterohexameric complex (FlhD_4_C_2_) that initiates transcription from promoters in the second level of hierarchy (25). FlhDC-family regulators appear in controlling gene expression of tra operons in SXT/R391family ICEs and IncA/C plasmids as well (26,14). These conjugative systems have similar regulatory mechanisms, but the DNA motifs recognized by the respective activator proteins, SetCD and AcaCD, are not related to each other or to that of the flagellar systems (27,28,14). SetCD and AcaCD activate the expression of conjugative gene clusters, in addition SetCD stimulates *int* and *xis* genes required for excision and integration of the ICE via conjugal transfer (27). The master regulator genes are controlled in SXT by SetR, a λ cI-like repressor that appears to be a sensor of DNA damage and host's SOS response signals (29), and two unrelated repressors, a Ner-like and a H-NS-like DNA-binding protein (Acr1 and Acr2), in IncA/C plasmids, respectively (14).

Unlike ICEs, IMEs are not self-transmissible elements, thus they need a different regulatory principle to ensure their effective transfer and long term stability. In this work, we describe how SGI1 exploits the regulatory mechanism of the conjugation system in IncA/C family plasmids for timing its excision, a crucial step in the transfer. We show that the plasmid-borne master activator binds to SGI1 *xis* promoter region, activating the excision and also leading to destabilization of the island. The promoters of *int* and *xis* have been defined and the binding site of the activator has been determined by footprinting and deletion analysis. We also proved that expression of *xis* is activator-dependent while *int* is constitutively expressed, and this regulatory model is presumably responsible for the efficient transfer and stable maintenance of SGI1. Furthermore, we have found an *flhDC*-like regulator encoded by SGI1. Its activity and possible role are also discussed.

## MATERIALS AND METHODS

### DNA and microbial techniques

Standard molecular biology procedures were carried out according to (30). Total DNA was prepared as described previously (31). Test PCRs were carried out as described (7). In standard PCR tests the following primer pairs were used: LJ2 – RJ2/RJ4 for attP, attsgi1for – C9-L2 for attB_ST_, attsgi1for – attsgi1rev for attB_Ec_, attsgi1for – LJ2 for DRL, RJ2/RJ4 – C9-L2 for DRR_ST_, and RJ2/RJ4 – attsgi1rev for DRR_Ec_. PCR random mutagenesis, electrophoretic mobility shift assay (EMSA), footprinting and primer extension assays are described in Text S1–S3. Oligonucleotides are listed in Supplementary Table S1. Electroporation was carried out using BTX Electro Cell Manipulator 600 with 2-mm gap electroporation cuvettes as described (31). Gene KO experiments were carried out by the one-step recombination method (32) using the λ Red recombinase producer plasmid pKD46 or its Gm^R^ derivative pJKI648 and pKD3 template plasmid for amplification of the gene KO fragments (Text S4). Oligonucleotide primers for gene KO amplicons were designed according to the published sequences of pP99–018 (GenBank: AB277723) and SGI1 (GenBank: AF261825). Bacterial strains (Supplementary Table S2) were routinely grown at 37°C in LB supplemented with the appropriate antibiotics used at a final concentration as follows: ampicillin (Ap) 150 μg/ml, chloramphenicol (Cm) 20 μg/ml, kanamycin (Km) 30 μg/ml, spectinomycin (Sp) 50 μg/ml, streptomycin (Sm) 50 μg/ml, nalidixic acid (Nal) 20 μg/ml, gentamicin (Gm) 25 μg/ml, tetracycline (Tc) 10μg/ml. For maintaining and curing the plasmids with temperature-sensitive pSC101 replication system 30 and 42°C incubation temperatures were applied, respectively. Standard conjugation assays were carried out in 4–6 replicates as described (7). For β-galactosidase drop tests β-gal tester constructs and one of the plasmids expressing the activator genes (if required) were transformed into TG1 cells, transformant colonies were grown to a mid-log phase under selection for both plasmids in LB + Km + Sm and then 3 μl culture was dropped onto LB + Km + Sm plates supplemented with 0.004% X-gal. β-Galactosidase assays were carried out according to (33) except that cultures were grown in LB+antibiotics at 37°C to OD_600_ ∼0.3. AcaCD expression from pJKI888 was induced with 0.05 mM IPTG.

### Plasmid constructions

Relevant features of plasmids are listed in Supplementary Table S3, while detailed methodology of plasmid constructions is described in Text S5.

### SGI1 segregation tests

For monitoring the segregation of SGI1 from *S*. Typhimurium strains, single colonies were picked from LB plates supplemented with appropriate antibiotics (Tc for SGI1 and Gm for R55, if present) and grown to a stationary phase (ca. 10^9^ cfu/ml) in LB medium at 37°C without antibiotic selection for SGI1. Subsequently 10 μl cultures were transferred into 3 ml fresh medium and grown again to stationary phase. This process was repeated 10 times (two passages per day, each passage represented ca. eight generations). Cultures from the first and tenth passages were spread onto LB plates in 10^5^× dilution and replica plated onto LB and LB+Tc plates to count Tc^S^ segregants. All Tc^S^ colonies from strains lacking R55 were tested for the other SGI1 resistance markers (Cm, Sm/Sp and Ap), while those having R55 were tested for Sm^R^/Sp^R^ (Cm^R^ and Ap^R^ markers are common with R55). Colony PCRs specific for DRL, DRR_ST_, attB_ST_ and the inner segment of SGI1 (using primer pairs S025rev–S026for) were carried out to verify the absence of the island.

The segregation assay with TG1Nal::SGI1-C containing plasmids that express *acaCD* genes (pJKI813, pJKI816, pJKI828, R55) and pJKI691 (negative control), respectively, was carried out as described above except the starter cultures were grown in LB + Km + Sm + Sp and passages were made in LB + Km. Total cell count was determined from the first and fifth passages on LB+Km plates in three replicates, while the number of SGI1^−^ colonies were determined by replica plating onto LB + Sm + Sp. The lack of SGI1 in randomly selected set of Sm^S^Sp^S^ segregants was verified by colony PCRs specific for attB_Ec_ and DRL.

The FlhDC_SGI1_-mediated SGI1-C segregation was monitored by transforming expression vectors pJKI878 (*acaCD*), pGMY3 (*flhDC_SGI1_*) and pET16b (negative control), respectively, into Tuner::SGI1-C strain and replica plating the Ap^R^ transformants directly onto LB+Ap and LB+Sm+Sp plates.

### Construction and conjugation tests of strains harbouring SGI1-C::*oriT*_RK2_

To simplify the KO and conjugation experimental setup we applied SGI1-C, which contains intact SGI1 backbone and a reduced MDR region (Sm^R^/Sp^R^, Sul^R^) due to a deletion in the In104 region (7). *OriT*_RK2_ was inserted into *S026* by single gene KO method (Text S4). R55 or pRK2013 was then conjugated into the three *Salmonella* strains carrying either SGI1-C or their SGI1-C::*oriT*_RK2_ derivatives. The transconjugants were used in attP specific PCRs and conjugation assays as donor strains to test the ability of SGI1-C::*oriT*_RK2_ for excision and conjugative transfer. Transfer frequencies obtained with *E. coli* TG90Nal recipient were expressed as transconjugant per recipient titers from three to five replicates. SGI1-C::*oriT*_RK2_ transconjugants were isolated following overnight cultivation of the conjugation mixtures under selection for the markers of SGI1-C transconjugants (SmTcNal).

SGI1-C::*oriT*_RK2_ was transferred from ST21S1::*oriT*_RK2_/R55 into *E. coli* S17–1Nal carrying the whole transfer apparatus of RK2 integrated onto the chromosome (34) to test the complementation by AcaCD regulator.

## RESULTS

### Identification of R55-encoded genes responsible for excision and high frequency loss of SGI1

Segregation tests demonstrated that SGI1 is very stable in *Salmonella* Typhimurium DT104 strains lacking IncA/C plasmid R55 (7), while it is lost at a high rate when R55 is present (Table 1). All colonies obtained from strains lacking R55 proved to be SGI1^+^ even after 10 passages, including the five Tc^S^ colonies that proved to harbour SGI1-B or SGI1-C (Supplementary Figure S2AB). In contrast, strains containing R55 produced ca. 4–7% Tc^S^ colonies at the first passage and their ratio even exceeded 46% in one strain at the tenth. Resistance pattern and PCR tests (Supplementary Figure S2A) carried out on randomly selected Tc^S^ colonies proved the loss of the whole island. PCRs monitoring attP (formed by the excised and circularized SGI1) and attB (the empty site left behind the excised island, Supplementary Figure S1A) that were carried out on total DNAs from cultures of the first passage showed elevated excision activity in the presence of R55 in all tested strains (Supplementary Figure S1B). Based on these observations, we presumed that high frequency loss of SGI1 was due to the increased rate of excision promoted by R55. In order to test whether the enhanced attP formation is the result of the conjugative transfer process or a specific function encoded by R55, we inserted the *oriT* region of RK2 plasmid into SGI1-C, thus the island became potentially mobilizable by the RK2 derivative plasmid pRK2013 (35). The attP specific PCRs and standard conjugation assays showed that, in contrast to R55, pRK2013 could not promote detectable excision and transfer of SGI1-C::*oriT*_RK2_ (Supplementary Figure S1C, Figure 1B). The SGI1-C::*oriT*_RK2_ transconjugants, that could be observed only by selective cultivation of the conjugation mixtures, derived presumably from pRK2013-dependent transfer of the spontaneously excised island (7). These results suggested that an R55-specific function, missing from pRK2013, is essential for the high frequency transfer of SGI1. The genes responsible for this function were determined (Supplementary Figure S3, Text S6) and the database search revealed that the two orfs belong to the *flhDC* master regulator family. Following the recent publication of the key activator of transfer genes in IncA/C plasmids (14) they turned out to be identical to *acaCD*.

**Table 1.** SGI1 loss from *S*. Typhimurium DT104 strains in the presence of IncA/C plasmid R55

No. of passage	Strain	Total no. of colonies	Tc^S^ colonies	Rate of SGI1^−^ colonies (%)
1	ST1369	854	0	<0.12
	ST1375	772	1^a^	<0.13
	ST1773	1160	0	<0.09
	ST1369/R55	490	33	6.7
	ST1375/R55	1132	49	4.3
	ST1773/R55	428	27	6.3
10	ST1369	563	2^b^	<0.18
	ST1375	568	1^b^	<0.17
	ST1773	487	1^b^	<0.21
	ST1369/R55	700	327	46.7
	ST1375/R55	847	61	7.2
	ST1773/R55	588	174	29.6

^a^Sm^R^/Sp^R^ derivative (SGI1-C variant).

^b^Ap^R^ derivative (SGI1-B variant).

Elimination of *aca*CD genes from the helper plasmid (Text S4) led to the complete loss of both self-transfer and mobilization of SGI1-C, while trans complementation by *aca*CD restored the excision of the island and conjugation of both the helper plasmid and SGI1-C (Figure 1C). *Aca*CD alone was also able to complement the mobilization failure of SGI1-C::*oriT*_RK2_ by the transfer apparatus of plasmid RK2 (Figure 1D). All plasmid constructs encoding *aca*CD caused SGI1 excision in TG1Nal::SGI1-C (Supplementary Figure S3B) as was also observed by (14) and also induced frequent loss of SGI1 on the population level depending on the expression levels of the activator (Supplementary Table S4). In the most extreme case, when the plasmid containing *aca*CD fused to P_tac_ (pJKI888) was transformed into TG1Nal::SGI1-C very few transformants were obtained and they could not be maintained under selection for both the plasmid and SGI1-C. This phenomenon was reminiscent of some kind of incompatibility. These observations clearly suggest that orfs *aca*CD are responsible for both the excision initiation and high rate of SGI1 loss.

### Determination of target site of *acaCD* regulator on SGI1

**Figure 2.**
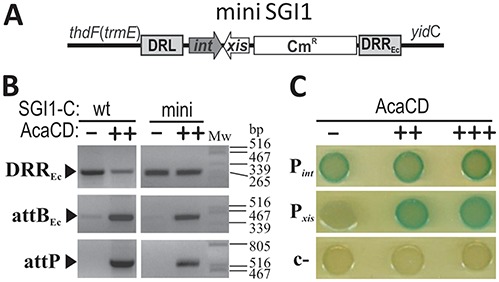
Localization of AcaCD target region in SGI1. (**A**) Schematic representation of mini SGI1 residing on the *E. coli* chromosome. (**B**) AcaCD-induced excision of wt SGI1-C (wt) and miniSGI1 (mini) in TG1Nal strain. The activator was expressed from pJKI828 (++). Vector pJKI691 (−) was used as negative control. (**C**) AcaCD activates expression from the upstream region of *xis* gene. Non-coding upstream regions of *int* and *xis* were fused with a promoterless *lac*Z gene in β-gal tester plasmids. Constructs pJKI995 (P*_int_*), pJKI1003 (P*_xis_*) and pJKI990 (c−) were monitored for *lacZ* expression in the presence or absence of AcaCD by drop test. The activator was expressed from its own promoter (pJKI828, ++) or P_tac_ (pJKI888, +++). Vector pJKI691 (−) was used as negative control.

The above results and the fact that the *int* and *xis* genes of SXT are regulated by *set*CD, an *flh*DC-family regulator, suggested that the *acaCD* genes may have similar function on SGI1. The backbone of SGI1 also carries the *int* and *xis* genes responsible for excision and integration (9) and several orfs that might be involved in SGI1 transfer (*S005*/*traN, S011-S012*/*traG, traH*). Since we have isolated a deletion mutant of SGI1-C called d1 (7), in which *S005–S012* region is missing without negative effect on the mobilization of the island, we hypothesized that *int* and/or *xis* genes can be the targets of AcaCD. To test this assumption, a mini SGI1 retaining only the *int* and *xis* genes with their upstream regions was constructed in TG1Nal::SGI1-C (Figure 2A). AttP specific PCR showed that the mini SGI1 can excise in the presence of *acaCD* genes similarly to wt SGI1-C (Figure 2B). Upstream regions of *int* and *xis* genes potentially containing the *cis* regulatory elements were assayed by β-galactosidase drop tests to examine whether the genes are under the control of AcaCD. Results showed that P*_int_* drives *lac*Z expression even without AcaCD, while P*_xis_* was only active in its presence (Figure 2C) leading us to the conclusion that AcaCD acts as an activator of *xis* gene.

#### Analysis of P_int_ promoter

**Figure 3.**
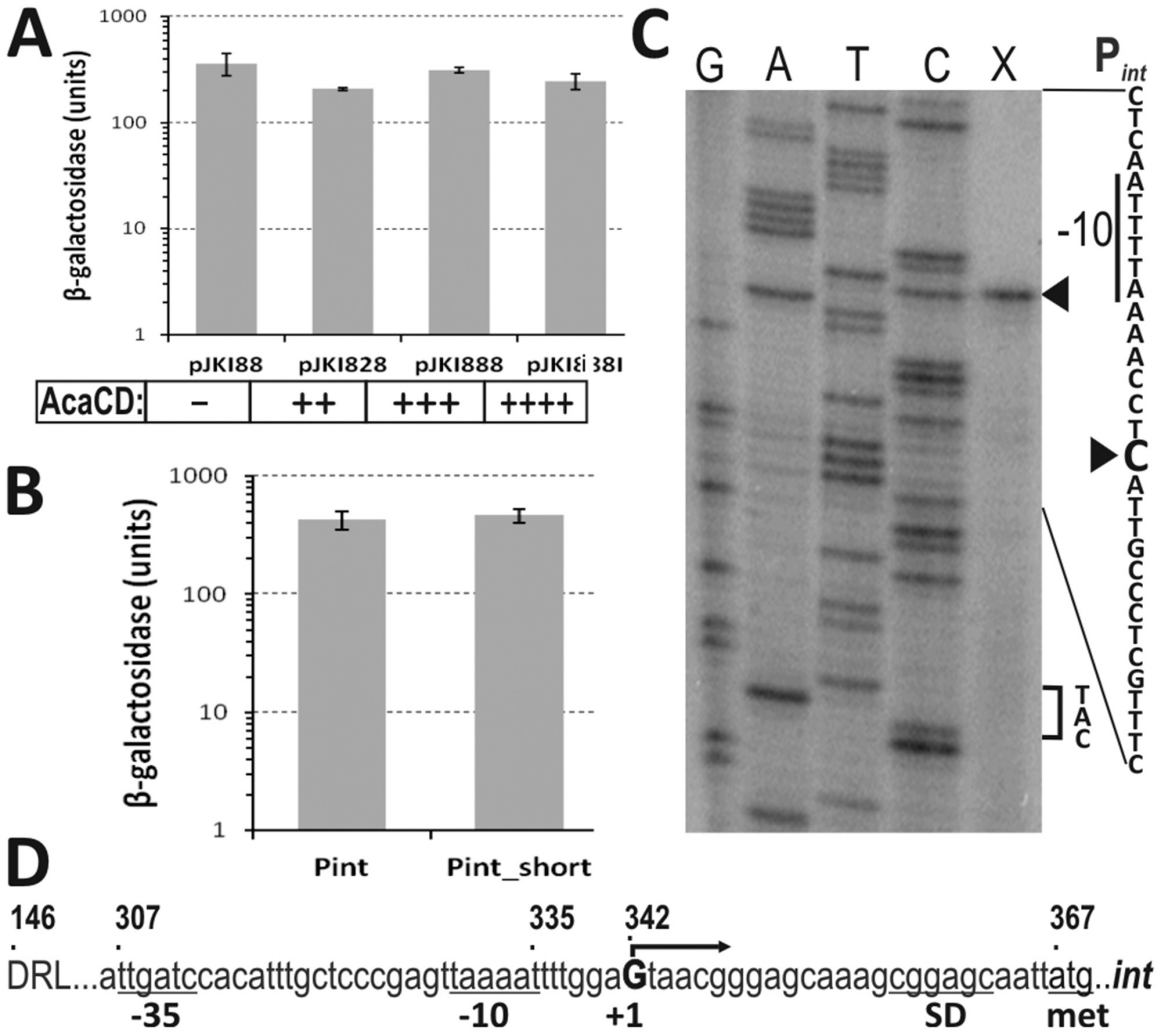
Analysis of promoter region P*_int_*. (**A**) The effect of AcaCD on the expression from P*_int_* region. The β-galactosidase assay was carried out with TG1 strain containing the tester plasmid pJKI995 (P*_int_*) and AcaCD producer plasmids pJKI828 or pJKI888, respectively. Vector pJKI88 lacking *acaCD* genes was used as negative control. Producer plasmid containing P_tac_ (pJKI888) was measured under non-inducing and inducing (pJKI888i) conditions. The relative expression levels of *acaCD* are indicated. (**B**) β-Galactosidase assay of the full length (pJKI863, 146–366 bp) and truncated (pJKI870, 269–366 bp) P*_int_* region. (**C**) Determination of *int* TSS. Primer extension reaction (lane X) was performed using total RNA purified from *E. coli* TG90 carrying pJKI995 and primer pUCfor21 annealing near to the start codon of *lac*Z gene. Lanes G, A, T, C: Sanger sequencing reactions obtained using pUCfor21 and pJKI995 template DNA. Arrowheads point to the C base on the non-transcribed strand corresponding to the G located 25 bp upstream from the ATG codon on the sense strand. The −10 box and the start codon are indicated. (**D**) Promoter region of *int*. Coordinates above the sequence refer to published SGI1 sequence (AF261825). The start codon, deduced Shine-Dalgarno, −10, −35 boxes and TSS of *int* are indicated.

The drop test and β-gal assay proved that AcaCD has no effect on the activity of P*_int_* (Figure 3A) suggesting that *int* is expressed constitutively. In order to identify promoter P*_int_*, upstream region of *int* was reduced to 98 bp (P*_int_*_short), which did not affect its promoter activity (Figure 3B). To examine the putative promoter boxes, random mutagenesis was carried out by suboptimal PCRs (Text S1) amplifying the 98 bp upstream region. Sequence alignment showed three regions where mutations accumulated (Supplementary Figure S4). Microdeletions and a single A→T change 4–8 bp upstream from the start codon indicated the potential SD-element, T→C and A→G base changes 57–60 bp predicted the putative -35 box, and a single A→G mutation affected the putative −10 box. The startpoint of *int* transcript (TSS) was located 25 bp upstream of the start codon of *int* gene by primer extension experiment (Figure 3C). The predicted promoter at 307–334 bp of SGI1 (Figure 3D) matches well to the consensus σ^70^ promoter and drives *int* expression constitutively.

#### Analysis of P_xis_ promoter

**Figure 4.**
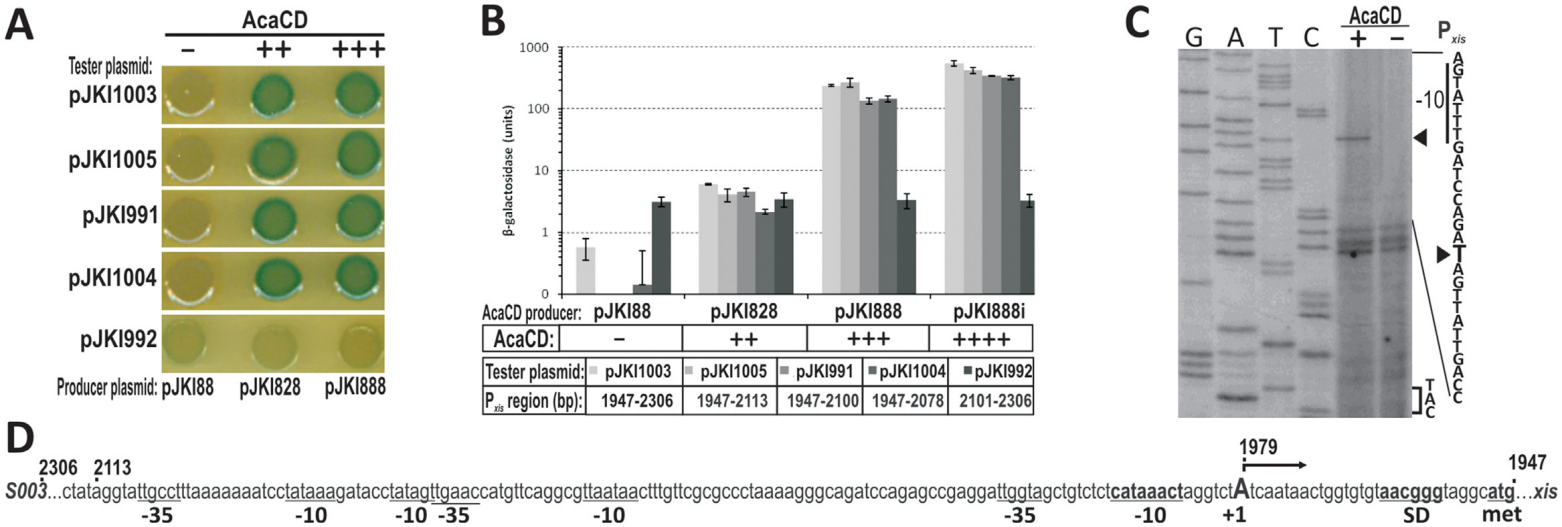
Analysis of promoter region P*_xis_*. (**A**) Drop test of different regions of P*_xis_* in the presence of AcaCD. The assay was carried out with TG1 strain containing the tester plasmids pJKI1003, pJKI1005, pJKI991, pJKI1004 and pJKI992 and AcaCD producer plasmids pJKI828 or pJKI888, respectively. Coordinates of P*_xis_* regions are shown in panel B (see also Supplementary Figure S5). Vector pJKI88 was used as negative control. (**B**) β-Galactosidase assay of different regions of P*_xis_* in the presence of AcaCD. Experiment was carried out as described in Figure 3. (**C**) Determination of *xis* TSS. Extension reactions were performed using primer pUCfor21 and total RNA purified from *E. coli* TG90 carrying tester plasmid pJKI1003 ± AcaCD producer plasmid pJKI888 (lanes + and −). Lanes G, A, T, C: Sanger sequencing reactions obtained using pUCfor21 and pJKI1003 template DNA. Arrowheads point to the T base on the non-transcribed strand corresponding to the A located 28 bp upstream to the ATG codon on the sense strand. The putative −10 box and the start codon are indicated. The presence (+) or absence (−) of AcaCD is shown. (**D**) Proximal fragment of *xis* promoter region. Coordinates above the sequence refer to published SGI1 sequence. The startpoint of *xis* transcript (uppercase A) and the deduced Shine-Dalgarno, −10 boxes are in bold, other potential −10 and −35-like elements are also indicated.

The non-coding region located between *xis* and orf *S003* contains at least three promoter-like elements, however, none of them has optimal spacing between the putative −35 and −10 boxes (Supplementary Figure S5). The functional promoter and the target region of AcaCD were localized in β-gal drop test and assay. Drop test revealed that without AcaCD even the full length P*_xis_* region was not able to drive *lac*Z expression indicating that there is no true constitutive promoter in this region, while in the presence of the activator expression was efficient from all proximal fragments of P*_xis_* (Figure 4A).

In the β-gal assay similar levels of expression were observed with the proximal fragments and the full length P*_xis_* region, and promoter activity was dependent on AcaCD concentration (Figure 4B). Compared to the negative control 10–20× activity was observed when AcaCD was expressed from its own promoter, while expression from P_tac_ resulted in ca. 300–400× (without induction) and ca. 700–900× (with induction) increase. The distal fragment, on the other hand, had a basal promoter activity that appeared completely independent of AcaCD (Figure 4B) and might account for the native *xis* expression in SGI1 (11). The observations suggested that even the shortest proximal fragment (pJKI1004) contains all the *cis* elements required for the regulation of *xis* expression.

For further specification of P*_xis_* TSS was determined in primer extension experiment. The startpoint of *xis* transcript was detectable only in the presence of AcaCD and localized 28 bp upstream of the start codon (Figure 4CD). The nearest putative −10 and −35 motifs found upstream from the TSS are unlikely to constitute an active promoter due to the too short spacing, which is supported by the observation that this region had no promoter activity in absence of AcaCD. We supposed that *xis* transcription requires the binding of AcaCD in the proximal 127 bp of P*_xis_* region.

#### Identification of the binding site of AcaCD activator in P_xis_

To specify the binding site of AcaCD in P*_xis_* region, a purification method was developed for the activator protein. Our first approach to purify AcaC and AcaD subunits separately was unsuccessful due to the denaturation of C subunit in the absence of D. Similar phenomenon was observed in the case of *E. coli* FlhC (36). To overcome this problem the two partially overlapping orfs *aca*CD were cloned and expressed as native D subunit and C-terminally tagged C subunit fused to an intein-chitin binding domain, respectively. During the purification procedure, only the C subunit was tethered to the chitin column and D associated to C via their native binding capacity (Supplementary Figure S6A). This purification method helped to maintain the regulator protein in its native and functional form and proved that the two polypeptides constitute a heteromeric complex.

**Figure 5.**
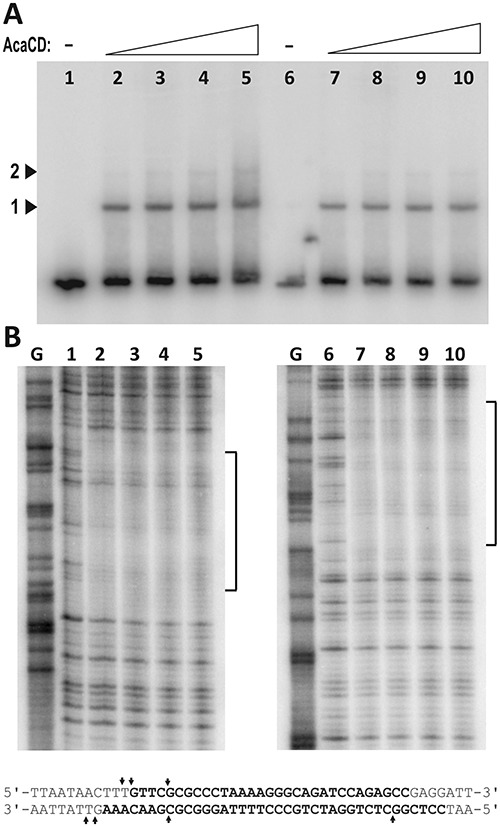
Investigation of DNA-protein interactions between P*_xis_* and AcaCD. (**A**) Detection of P*_xis_* DNA-AcaCD complexes by EMSA. ^32^P single-end-labelled proximal fragment of P*_xis_* (1947–2100 bp of SGI1) was subjected to AcaCD binding in 50 μl volume. Five μl reaction mix was applied for EMSA and the rest was used in the footprinting assay (panel B). DNA of P*_xis_* was labelled at EcoRI end in lanes 1–5 (corresponding to the upper strand in the footprinting experiments and the sequence in panel B), and at HindIII end in lanes 6–10 (lower strand), respectively. AcaCD content of the 50 μl binding reactions was: lanes 1 and 6, 0 μg; lanes 2 and 7, 5 μg; lanes 3 and 8, 10 μg; lanes 4 and 9, 20 μg; lanes 5 and 10, 30 μg. Arrowheads point to the primary (1) and higher order (2) complexes. (**B**) Determination of AcaCD binding site by DNaseI protection footprinting experiment. G, G-specific Maxam-Gilbert sequencing lane. Brackets indicate the regions protected by bound AcaCD, corresponding bases are in bold in the sequence below. Arrows indicate positions with enhanced DNaseI cleavage.

The interaction between the purified AcaCD protein and different fragments of P*_xis_* was first investigated by EMSA. The 154 bp proximal region of P*_xis_* bound specifically to the protein, and was used to optimize binding conditions. A primary shifted band appeared upon addition of AcaCD, while increasing amount of protein resulted a second, slower migrating band, too, representing higher-order complexes, most likely sandwich of two P*_xis_*–AcaCD complexes (Figure 5A). These complexes were already detectable when unbound DNA was still available. Addition of >30 μg protein to the binding reactions resulted in smearing due to formation of possibly non-specific DNA–protein complexes by aggregation (not shown).

The binding site of AcaCD in P*_xis_* region was located by DNaseI footprinting assay. The protein–DNA contact area was ca. 40 bp on both strands 31 bp upstream of the TSS of *xis* (Figure 5B). In DNaseI footprinting the protected area is usually longer than the actual binding site that could be determined by shortening this region with four and six bases at the 5′ and 3′ sides, respectively (37), however, strict application of this rule may result in underestimating the length of the binding site and losing some binding abilities (38). Unusually, the protected P*_xis_* region in the lower strand appeared to be 3–4 bp longer on both sides than in the upper strand. Further studies are needed to determine whether the heteromeric AcaCD protein, a bent DNA-structure in the P*_xis_*–AcaCD complex or both are responsible for this phenomenon.

**Figure 6.**
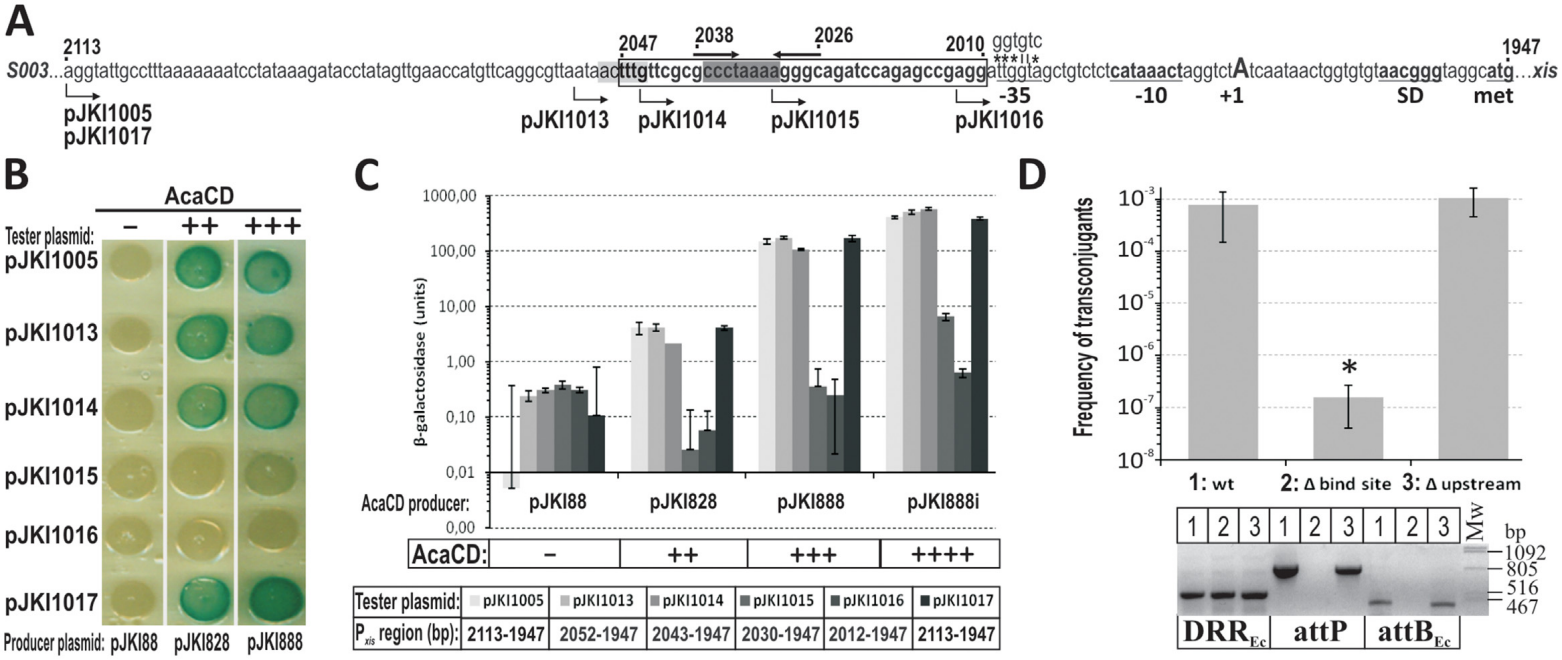
Mutation analysis of AcaCD binding region in P*_xis_*. (**A**) The proximal fragment of P*_xis_* region. The segment protected by AcaCD is boxed and shown in bold, the inverse repeat is indicated by thick arrows above the sequence, while the two sequence motifs repeated in the distal region are highlighted by light and dark grey (see also Supplementary Figure S5). Arrows below the sequence show the 5′ ends of P*_xis_* fragments in the tester plasmids. In pJKI1017 the putative −35 box was replaced for ‘ggtgtc’ sequence, mismatches are indicated by asterisks. Other symbols are as in Figure 4. (**B**) Promoter activity of truncated/mutated P*_xis_* regions. The relative expression levels of *acaCD* is indicated. Producer plasmids pJKI828, pJKI888, pJKI88 and the experimental setup are described in Figure 4. (**C**) β-galactosidase assay of the truncated/mutated P*_xis_* regions in the presence of AcaCD. Symbols and the experimental setup are described in Figures 3 and 4. The coordinates of P*_xis_* fragments inserted into the tester plasmids are shown below the plasmid names. (**D**) The effect of deletions on the excision and transfer frequency of SGI1-C. Two deletions were made in P*_xis_* region of a chromosomally integrated SGI1-C. In ‘Δbind site’ mutant the AcaCD-protected sequence and the distal segment of P*_xis_* region (2306–2013 bp) was removed. In ‘Δupstream’ mutant the binding site is intact and the deletion removed only the upstream sequence (2306–2053 bp). Excision and transfer of the deletion mutants were tested in the presence of R55. Transfer frequencies are expressed as transconjugant per TG2 recipient cfu. *Transfer frequency of ‘Δbind site’ mutant was under the detection limit. The image below the graph shows the DRR_Ec_, attP and attB_Ec_ specific PCRs for the strains applied in conjugation assay. Lane 1, TG1Nal::SGI1-C/R55; lane 2, TG1Nal::SGI1-CΔbind site/R55; lane 3, TG1Nal::SGI1-CΔupstream/R55.

The footprint area contains a perfect 5 bp inverse repeat (IR) separated by 3 bp spacing (GCCCTAAAAGGGC). The 7 bp CCCTAAA motif in the IR element and the 6 bp ACTTTG motif overlapping the 5′ end of protected area are repeated in direct orientation in the distal part of P*_xis_* region (Supplementary Figure S5). The possible functions of these repetitive elements were assayed by using β-gal tester plasmids carrying 5′ truncated parts of P*_xis_* promoter (Figure 6A). The functionality of the putative −35 box found at suboptimal length upstream the −10 motif was examined by using a tester plasmid where it was eliminated by transversions. Drop test showed that removing the whole or half of the protected region (pJKI1016, pJKI1015) destroyed *lac*Z expression even in the presence of excess AcaCD. The elimination of the −35 box (pJKI1017), or deletion of the region upstream the footprint area with or without the ACTTTG motif (pJKI1014, pJKI1013) had no significant effect on the function of the truncated P*_xis_* region (Figure 6B). The β-galactosidase assays gave similar results, however, removing the 6-bp motif caused a slight reduction in the activation efficiency if the activator was expressed from its own promoter (compare pJKI1013 and pJJKI1014), and a residual activity was also observed with the half binding site (pJKI1015) when AcaCD was present in large excess (Figure 6C). At last, the conjugal transfer and excision activities of SGI1-C deletion mutants affecting the distal P*_xis_* region and the footprint area were monitored in the presence of R55. Deletion of the distal region alone had no detectable effect, while the elimination of the distal region along with the binding site (2306–2012 bp) wiped out both activities (Figure 6D). Our results prove that the GCCCTAAAAGGGC IR motif located asymmetrically in the footprint area has a crucial role in AcaCD-dependent activation, and support the prediction that the 13 bp IR motif along with its 5′ 5-bp and 3′ 10-bp flanking sequences is the binding site of AcaCD in SGI1 P*_xis_* (14). It was also suggested that in AcaCD-dependent promoters the binding site is followed by a putative -35 box (14), however, elimination of the corresponding motif in P*_xis_* had no effect on the AcaCD-dependent activation (Figure 6C). This result hinted at P*_xis_* belongs to the class of -35-independent promoters, but the assumption was rejected since the −10 region of P*_xis_* does not show significant similarity to the ‘extended −10 promoters’ (39), and we rather believe that σ^70^ is recruited by the activator itself as it was suggested for SetCD and FlhDC (27,36). The promoter profile of P*_xis_* is consistent with that of the *tra* regulon of pVCR94ΔX (14) and the class II flagellar promoters of *E. coli* and *Salmonella* (40), supporting the assumption that P*_xis_* is activated via the class II pathway (41).

### SGI1 encodes for its own flhDC-like genes

**Figure 7.**
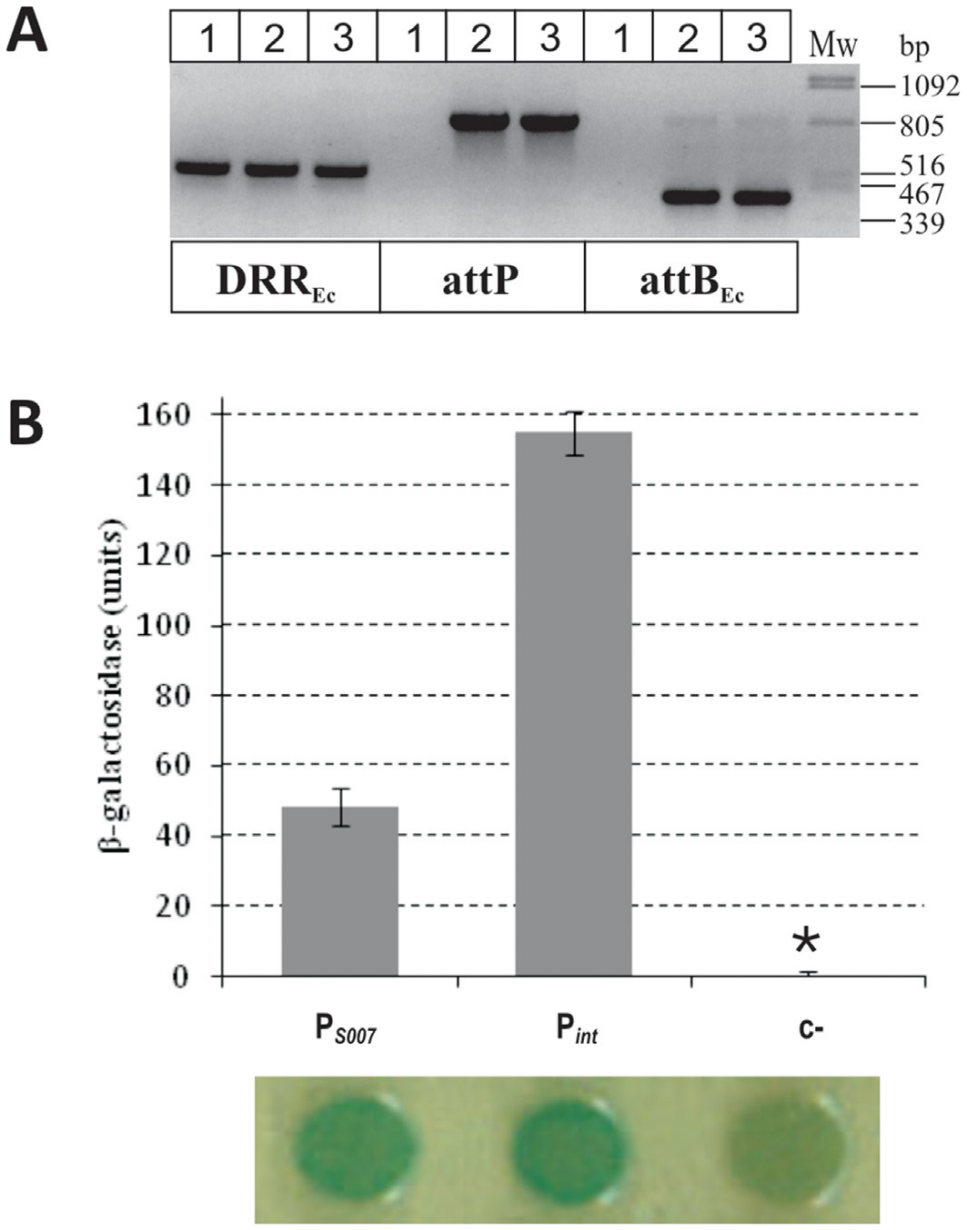
Validation of the activity of FlhDC_SGI1_ activator and promoter region P*_S007_*. (**A**) Detection of SGI1-C excision promoted by FlhDC_SGI1_. Colony PCRs specific for DRR_Ec_, attP and attB_Ec_ were carried out on Tuner::SGI1-C strain containing pET16b (lane 1, negative control), pGMY3 (lane 2, FlhDC_SGI1_) and pJKI878 (lane 3, AcaCD). Strains were grown in LB + Sm + Sp + Ap (selected for both SGI1 and the plasmid) without IPTG induction. (**B**) The promoter activity of *S007* upstream region in β-galactosidase assay and drop test. The activity of P*_S007_* carried on tester plasmid pMSZ945 (7626–8057 bp SGI1 segment) was compared to that of P*_int_* (pJKI995). Vector pJKI990 was used as negative control (c−). *β-Galactosidase expression of c− was around the detection limit.

Orfs *S006* and *S007* on SGI1 show high degree of homology to *acaCD* and lower level of relatedness to *set*CD of SXT and *flh*DC of *Salmonella* and *E. coli* (Supplementary Figure S7), suggesting that they are members of the *flh*DC-family. The native expression of both genes was reported (11), but their function has not yet been identified. Robust activation of SGI1 excision by *S006* and *S007*, designated as *flhC*_SGI1_ and *flhD*_SGI1_, respectively, seems unlikely as spontaneous loss of the island can not be observed (7) and excision is hardly detectable by PCRs (6,7,10,12). The role of *flhDC*_SGI1_ was first examined in TG1Nal::SGI1-C harbouring R55, where deletion of the two orfs had no detectable effect on conjugation frequency compared to the wt SGI1-C (wt: 3.2±1.6×10^−4^, Δ*S006-S007*: 5.0 ±4.1×10^−4^ per recipient cfu). The effect of excess FlhDC_SGI1_ on the stability of the island was then examined by measuring the transformation efficiency in Tuner::SGI1-C strain with an expression vector carrying *flhDC_SGI1_* fused to P_T7_ (Supplementary Figure S6B). Compared to the negative control (7.6 × 10^5^ cfu/mg DNA), FlhDC_SGI1_ producer plasmid resulted 1/3 (2.4 × 10^5^ cfu/mg DNA), whereas the analogous construct expressing AcaCD yielded 1/20 colonies (3.1 × 10^4^ cfu/ mg DNA). Transformant colonies gave strong PCR signal for attP and attB proving that, similarly to AcaCD, FlhDC_SGI1_ also induces excision (Figure 7A). Transformants obtained with the *acaCD* construct could not be maintained under selection for both the plasmid and SGI1-C even without induction (similar incompatibility-like phenomenon was observed with the plasmid expressed AcaCD from P_tac_ in strain TG1Nal::SGI1-C) in contrast to transformants expressing FlhDC_SGI1_. To compare the effect of the two FlhDC-like regulators on the stability of SGI1-C, transformants were selected only for the presence of the expression vectors. Replica plating of these colonies showed that AcaCD induced significantly higher rate of SGI1-loss (97–100%) than FlhDC_SGI1_ (0.4–4.4%), while the control plasmid had no detectable effect (<0.14%). These data suggest that FlhDC_SGI1_ can act similarly to its plasmid-encoded counterpart when provided from an expression vector, but it had much weaker effect on the stability of the island. Finally, the promoter activity of *S007* upstream region was examined in β-galactosidase drop test and assay, which showed that this region contains a constitutive promoter with an activity of ca. 1/3 of P*_int_* (Figure 7B). The observations suggest that FlhDC_SGI1_ can act as a functional regulator, but it is less effective in promoting SGI1-loss than AcaCD and its native promoter is less active than P*_int_*, which might explain why neither excision nor segregation of SGI1 was observed without overexpression of FlhDC_SGI1_.

## DISCUSSION

In the lack of autonomous plasmid-like replication GIs cannot stably exist extrachromosomally, thus they need to be integrated into the host's chromosome. The vertical transmission of GIs is ensured by the integration, while the horizontal dissemination needs their excision from the chromosome, followed by conjugal transfer and integration into the recipient. Different control logics have been evolved in the two major groups of GIs, ICEs and IMEs, to regulate the excision–transfer–integration cycles. Unlike ICEs, IMEs can not conjugate autonomously, thus their excision has to be synchronized with the presence of their transfer-competent helper. In this work we describe how SGI1 solves this issue by exploiting the conjugal control mechanism of the IncA/C helper plasmids. We demonstrate that AcaCD, the key regulator of IncA/C transfer (14), also acts as the activator of SGI1 excision, which is indispensable for the efficient transfer of the island. The activator-dependent induction of excision leads to destabilization of SGI1, which is reminiscent of an incompatibility between SGI1 and IncA/C plasmids, and it may explain the observation that co-acquisition of the island and its helper in the recipients is less frequent than expected (14,27).

Excision, which produces the mobilizable circular form of SGI1, is catalysed by Int and Xis (10), thus the expression regulation of these proteins is a key factor in SGI1 dissemination. Infrequent spontaneous excision (6,7,10,12) ensures very low level of conjugal transfer as was observed with SGI1-C::*oriT*_RK2_ (Supplementary Figure S1BC), however, the RK2-based system was efficiently complemented *in trans* by providing AcaCD, which induced SGI1 excision (Figure 1D). It has been shown that AcaCD is required for the expression of all plasmid-borne transfer-related genes (14), but our results prove that the master regulator is also necessary for activation of excision, and therefore, SGI1 transfer. These observations suggest that excision is probably the major limiting factor in SGI1 conjugation.

AcaCD induces SGI1 excision via transcriptional activation of Xis expression, which involves binding the master regulator to the upstream region of *xis*. The AcaCD target site was identified by EMSA and footprint experiments, which confirmed the binding sequence predicted recently (14). Mutation analysis revealed that the highly conserved 13 bp IR motif located asymmetrically in the protected region (Figure 6A) has a crucial role in AcaCD-dependent activation. Unlike P*_xis_*, the *int* promoter is not preceded by potential AcaCD binding sites and it seems to function constitutively. Since the predicted AcaCD binding sites are located in the first third of SGI1 and directed towards the 5′ end of the island (14), AcaCD-dependent activation of P*_int_* through attP in the circular form of SGI1 seems also unlikely. This manner of *xis* and *int* regulation enables SGI1 to hijack the IncA/C helper plasmids and ensures integration and stable maintenance in the recipient in the absence of helper plasmid. In contrast to SXT-family ICEs that require the SetCD-induced expression of *xis* and *int* both in the donor and recipient cells to fulfil the excision-transfer-integration cycle (27), SGI1 expresses *int* constitutively at a relatively high level (Figure 3A) (11), while *xis* is almost silent in absence of AcaCD (Figure 4B). Although Int is able to excise SGI1, the process is inefficient in absence of Xis (10), thus the recombination activity of Int is pushed towards integration, which can explain the low excision rate and high stability of SGI1 (7).

Due to AcaCD-dependent nature of transcriptional activation, P*_xis_* acts as a sensor of helper plasmid entry. Although *acaCD* expression is regulated by two different repressors (14), the activator is still expressed at a level that can be immediately ‘detected’ by P*_xis_*, thus triggering excision. This sensor mechanism is probably very sensitive as it was indicated by the excision tests carried out with promoterless *acaCD* constructs (Supplementary Figure S3AB). The presumed sensory function of P*_xis_* is to produce the transfer competent circular form of the island at the optimal time. In the presence of helper the high rate of excision causes remarkable destabilization of SGI1 (Table 1, Supplementary Table S4), but on evolutionary time-scale this seems to be ‘affordable cost’ for the benefits of dissemination. Following conjugal transfer Xis expression drops to the basal level in the absence of activator, and SGI1 is integrated by Int synthesized *de novo* from its constitutive promoter. If the recipient already contains the helper plasmid the integration is less efficient (unpublished observations), undoubtedly due to the permanent induction of Xis expression, which can also account for the low frequency co-acquisition of SGI1 and helper plasmid (14,27).

SGI1 encodes for its own FlhDC-family regulator, which is the closest known homologue of AcaCD (Supplementary Figure S7). FlhDC_SGI1_ shows the same activities as its plasmid-borne counterpart, but has much weaker effect on SGI1 stability. The main questions are why SGI1 is so stable in the presence of FlhDC_SGI1_ under native conditions (7) and what the function of this regulator is. The relatively low expression level of FlhDC_SGI1_ and its reduced activity to induce excision may explain the weaker destabilizing effect on SGI1 than observed with AcaCD. One can hypothesize that the excision inducing activity of FlhDC_SGI1_ is counterbalanced by the efficient reintegration by Int, however, the participation of an as-yet-unknown addiction system in SGI1 stability, similarly to SXT (42), can not be excluded either. The function of FlhDC_SGI1_ has to be elucidated. Deletion of its genes from SGI1-C had no detectable effect on the efficiency of mobilization, suggesting that it has no significant function in SGI1 dissemination. According to one possible scenario *flhDC*_SGI1_ is a remnant from an earlier evolutionary stage of the island when the ancient SGI1 was an ICE and FlhDC_SGI1_ fulfilled similar functions as SetCD does in SXT. Likewise, the predicted AcaCD binding sites on SGI1, which are located in front of *xis* and several conjugation-related genes (14), might be derived from target sites of FlhDC_SGI1_. Having lost the ability for self-transfer, FlhDC_SGI1_ probably became a destabilizing agent due to its excision promoting activity, which was silenced to the present level by mutations affecting the coding sequences and/or the promoter region. On the other hand, efficient excision and mobilization of the island presumably required fine-tuning the target site in P*_xis_* to bind AcaCD, possibly resulting a more attractive binding site for AcaCD than FlhDC_SGI1_ itself. Since FlhDC_SGI1_ is highly similar to AcaCD, there might be relationship between their binding sites, too, which could have enabled the rapid mutational transition toward hijacking the IncA/C encoded regulator. The ongoing investigations of this issue may shed light on the evolution of SGI1-family of IMEs.

## Supplementary Material

SUPPLEMENTARY DATA

## References

[1] Daccord A., Ceccarelli D., Rodrigue S., Burrus V. (2012). Comparative Analysis of Mobilizable Genomic Islands. J. Bacteriol..

[2] Douard G., Praud K., Cloeckaert A., Doublet B. (2010). The Salmonella genomic island 1 is specifically mobilized in trans by the IncA/C multidrug resistance plasmid family. PLoS One.

[3] Chun J., Grim C.J., Hasan N.A, Lee J.H., Choi S.Y., Haley B.J., Taviani E., Jeon Y.-S., Kim D.W., Lee J.-H. (2009). Comparative genomics reveals mechanism for short-term and long-term clonal transitions in pandemic Vibrio cholerae. Proc. Natl. Acad. Sci. U.S.A..

[4] Mulvey M.R., Boyd D.A, Olson A.B., Doublet B., Cloeckaert A. (2006). The genetics of Salmonella genomic island 1. Microbes Infect..

[5] Threlfall E.J. (2000). Epidemic Salmonella typhimurium DT 104 — a truly international multiresistant clone. J. Antimicrob. Chemother..

[6] Boyd D.A., Peters G.A., Ng L., Mulvey M.R. (2000). Partial characterization of a genomic island associated with the multidrug resistance region of Salmonella enterica Typhymurium. FEMS Microbiol. Lett..

[7] Kiss J., Nagy B., Olasz F. (2012). Stability, entrapment and variant formation of Salmonella genomic island 1. PLoS One.

[8] Hall R. (2010). Salmonella genomic islands and antibiotic resistance in Salmonella enterica. Future Microbiol..

[9] Boyd D., Peters G.A., Cloeckaert A., Boumedine K.S., Chaslus-Dancla E., Imberechts H., Mulvey M.R. (2001). Complete nucleotide sequence of a 43-kilobase genomic island associated with the multidrug resistance region of Salmonella enterica Serovar Typhimurium DT104 and its identification in phage type DT120 and serovar agona. J. Bacteriol..

[10] Doublet B., Boyd D., Mulvey M.R., Cloeckaert A. (2005). The Salmonella genomic island 1 is an integrative mobilizable element. Mol. Microbiol..

[11] Golding G.R., Olson A.B., Doublet B., Cloeckaert A., Christianson S., Graham M.R., Mulvey M.R. (2007). The effect of the Salmonella genomic island 1 on in vitro global gene expression in Salmonella enterica serovar Typhimurium LT2. Microbes Infect..

[12] Djordjevic S.P., Cain A.K., Evershed N.J., Falconer L., Levings R.S., Lightfoot D., Hall R.M. (2009). Emergence and evolution of multiply antibiotic-resistant Salmonella enterica serovar Paratyphi B D-tartrate-utilizing strains containing SGI1. Antimicrob. Agents Chemother..

[13] Doublet B., Golding G.R., Mulvey M.R., Cloeckaert A. (2008). Secondary chromosomal attachment site and tandem integration of the mobilizable Salmonella genomic island 1. PLoS One.

[14] Carraro N., Matteau D., Luo P., Rodrigue S., Burrus V. (2014). The master activator of IncA/C conjugative plasmids stimulates genomic islands and multidrug resistance dissemination. PLoS Genet..

[15] Johnson T.J., Lang K.S. (2012). IncA/C plasmids An emerging threat to human and animal health?. Mob. Genet. Elements.

[16] Fernández-Alarcón C., Singer R.S., Johnson T.J. (2011). Comparative genomics of multidrug resistance-encoding IncA/C plasmids from commensal and pathogenic Escherichia coli from multiple animal sources. PLoS One.

[17] Welch T.J., Fricke W.F., McDermott P.F., White D.G., Rosso M.-L., Rasko D.A, Mammel M.K., Eppinger M., Rosovitz M.J., Wagner D. (2007). Multiple antimicrobial resistance in plague: an emerging public health risk. PLoS One.

[18] Fricke W.F., Welch T.J., McDermott P.F., Mammel M.K., LeClerc J.E., White D.G., Cebula T.A., Ravel J. (2009). Comparative genomics of the IncA/C multidrug resistance plasmid family. J. Bacteriol..

[19] Carraro N., Sauvé M., Matteau D., Lauzon G., Rodrigue S., Burrus V. (2014). Development of pVCR94ΔX from Vibrio cholerae, a prototype for studying multidrug resistant IncA/C conjugative plasmids. Front. Microbiol..

[20] Doublet B., Boyd D., Douard G., Praud K., Cloeckaert A., Mulvey M.R. (2012). Complete nucleotide sequence of the multidrug resistance IncA/C plasmid pR55 from Klebsiella pneumoniae isolated in 1969. J. Antimicrob. Chemother..

[21] Aldridge P., Hughes K.T. (2002). Regulation of flagellar assembly. Curr. Opin. Microbiol..

[22] McCarter L.L. (2006). Regulation of flagella. Curr. Opin. Microbiol..

[23] Claret L., Hughes C. (2000). Functions of the subunits in the FlhD(2)C(2) transcriptional master regulator of bacterial flagellum biogenesis and swarming. J. Mol. Biol..

[24] Singer H.M., Erhardt M., Hughes K.T. (2013). RflM functions as a transcriptional repressor in the autogenous control of the salmonella flagellar master operon flhDC. J. Bacteriol..

[25] Wang S., Fleming R.T., Westbrook E.M., Matsumura P., McKay D.B. (2006). Structure of the Escherichia coli FlhDC complex, a prokaryotic heteromeric regulator of transcription. J. Mol. Biol..

[26] Burrus V., Waldor M.K. (2003). Control of SXT integration and excision. J. Bacteriol..

[27] Poulin-Laprade D., Matteau D., Jacques P.-É., Rodrigue S., Burrus V. (2015). Transfer activation of SXT/R391 integrative and conjugative elements: unraveling the SetCD regulon. Nucleic Acids Res..

[28] Wozniak C.E., Hughes K.T. (2008). Genetic dissection of the consensus sequence for the class 2 and class 3 flagellar promoters. J. Mol. Biol..

[29] Beaber J.W., Hochhut B., Waldor M.K. (2004). SOS response promotes horizontal dissemination of antibiotic resistance genes. Nature.

[30] Sambrook J., Fritsch E.F., Maniatis T. (1989). Molecular Cloning: A Laboratory Manual.

[31] Kiss J., Olasz F. (1999). Formation and transposition of the covalently closed IS30 circle: the relation between tandem dimers and monomeric circles. Mol. Microbiol..

[32] Datsenko K.A., Wanner B.L. (2000). One-step inactivation of chromosomal genes in Escherichia coli K-12 using PCR products. Proc. Natl. Acad. Sci. U.S.A..

[33] Miller J.H. (1972). Experiments in Molecular Genetics.

[34] Simon R., Priefer U., Pühler A. (1983). A broad host range mobilization system for in vivo genetic engineering: transposon mutagenesis in Gram negative bacteria. Bio/Technology.

[35] Figurski D.H., Helinski D.R. (1979). Replication of an origin-containing derivative of plasmid RK2 dependent on a plasmid function provided in trans. Proc. Natl. Acad. Sci. U.S.A..

[36] Liu X., Matsumura P. (1994). The FlhD / FlhC Complex, a Transcriptional Activator of the Escherichia coli Flagellar Class II Operons. J. Bacteriol..

[37] Suck D., Oefner C. (1986). Structure of DNase I at 2.0 Å resolution suggests a mechanism for binding to and cutting DNA. Nature.

[38] Papp P.P., Iyer V.N. (1995). Determination of the binding sites of RepA, a replication initiator protein of the basic replicon of the IncN group plasmid pCU1. J. Mol. Biol..

[39] Kumar A., Malloch R.A., Fujita N., Smillie D.A., Ishihama A., Hayward R.S. (1993). The minus 35-recognition region of Escherichia coli sigma 70 is inessential for initiation of transcription at an ‘extended minus 10′ promoter. J. Mol. Biol..

[40] Kutsukake K., Ohya Y., Iino T. (1990). Transcriptional analysis of the flagellar regulon of Salmonella typhimurium. J. Bacteriol..

[41] Browning D.F., Busby S.J. (2004). The regulation of bacterial transcription initiation. Nat. Rev. Microbiol..

[42] Wozniak R.A.F., Waldor M.K. (2009). A toxin-antitoxin system promotes the maintenance of an integrative conjugative element. PLoS Genet..

