# Supplementary material for: The master regulator of IncA/C plasmids is recognized by the Salmonella Genomic island SGI1 as a signal for excision and conjugal transfer
Source: Nucleic Acids Res. 2015 Oct 10;43(18):8735–45. doi: 10.1093/nar/gkv758 (PMC4605294; doi:10.1093/nar/gkv758)
Supplement: SUPPLEMENTARY DATA [file supp_43_18_8735__index.html]

The master regulator of IncA/C plasmids is recognized by the Salmonella Genomic island SGI1 as a signal for excision and conjugal transfer — SUPPLEMENTARY DATA 

# The master regulator of IncA/C plasmids is recognized by the *Salmonella* Genomic island SGI1 as a signal for excision and conjugal transfer

## SUPPLEMENTARY DATA

- SUPPLEMENTARY DATA
