# Supplementary material for: The master regulator of IncA/C plasmids is recognized by the Salmonella Genomic island SGI1 as a signal for excision and conjugal transfer
Source: Nucleic Acids Res. 2015 Oct 10;43(18):8735–45. doi: 10.1093/nar/gkv758 (PMC4605294; doi:10.1093/nar/gkv758)
Supplement: SUPPLEMENTARY DATA [file supp_gkv758_nar-01165-h-2015-File009.pdf]

Additional Materials and Methods and Supplementary data

Text S1. PCR random mutagenesis

For random mutagenesis of P_{int}, several rounds of suboptimal PCRs were performed in 1× DreamTaq buffer containing 2mM $MgCl_2$ and 0.2 mM of dNTP, 2 u DreamTaq, 0.2 µM of primers (sgi_promIntfor2 and sgi_promIntrev) and ~5 ng gDNA from ST21S1 as template and supplemented with (1a): 5 mM $MgCl_2$, 0,5 mM $MnCl_2$, 0,8 mM of dGTP and dCTP; (3b): 5 mM $MgCl_2$, 0,8 mM of dGTP and dCTP, (3c): 5 mM $MgCl_2$, 0,5 mM $MnCl_2$; (3d): 0,5 mM $MnCl_2$; (4e): 5 mM $MgCl_2$; (4f): 5 mM $MgCl_2$, 1,0 mM $MnCl_2$; (4g): 5 mM $MgCl_2$, 2,0 mM $MnCl_2$; (4h): 5 mM $MgCl_2$, 3,0 mM $MnCl_2$. PCR products were cloned into PstI-NcoI site of pJKI861 and transformed into TG1. Cells were spread onto LB+Sm+X-gal plates and plasmid DNA was isolated from white and light blue colonies for sequencing.

Text S2. Mobility shift assay and DNaseI footprinting

To obtain single-end-labelled EcoRI-HindIII fragment of pPP703, 2 µg plasmid DNA (QIAgen midiprep) was digested with EcoRI or HindIII, then end labelled with [α-^{32}P]dATP using 1 unit of Klenow polymerase (Fermentas) in fill-in reactions (30 min, 37 °C). After heat inactivation of the enzymes (20 min, 75 °C), DNA labelled at the EcoRI site was cleaved with HindIII, while DNA labelled at the HindIII site was cleaved with EcoRI and the labelled fragments were separated by electrophoresis in non-denaturing polyacrylamide gel and eluted from gel slices by the crush and soak method (1).

For column-based purification of AcaCD activator of R55 overnight culture of *E. coli* strain Tuner (DE3) (Novagen) transformed with pJKI958 was diluted100× in 500 ml fresh LB+Ap medium supplemented with 0.1 mM $ZnSO_4$ and grown to OD_{600} of 0.5 at 37 ºC. The culture was induced with 0.2 mM IPTG (8h, 30 ºC) under vigorous shaking, and harvested by centrifugation and frozen in two aliquots at – 70 ºC.

Bacteria from 250 ml induced culture were thawed on ice and resuspended in 20 ml buffer A (20 mM HEPES pH8.0, 50 mM KCl, 1 mM EDTA, 0.1 mM $ZnSO_4$, 10 % glycerol, 0.2% Triton X-100) supplemented with 600 µl of Complete protease inhibitor cocktail (Roche). Cells were disrupted using Emulsiflex C5 High Pressure Homogenizer device (Avestin) at 22-25 kpsi. The lysate was centrifuged (20 min, 4 ºC) and the cleared lysate (supernatant) was loaded at the flow rate of 0.2 ml/min onto 6 ml chitin column (NEB) equilibrated with buffer A. The bound protein was washed with 60 ml buffer A at the flow rate of 1 ml/min and then 6 ml buffer A+80 mM DTT was added to induce intein self-cleavage. Column was incubated O/N at room temperature and the harvested proteins were 5× concentrated using Amicon Ultra-4 Centrifugal filter unit (Millipore). Purified protein with the final concentration of 20.5 µg/µl was kept on ice until use.

DNA binding reactions and EMSA were carried out as follows: in addition to the end-labelled DNA (1-3 ng) and different amount of purified AcaCD protein (0-25 µg), each binding reaction (25 µl) contained 2.5 µl 10× binding buffer (200 mM Tris-HCl (pH 7.4), 500 mM KCl, 10 mM $MgCl_2$, 10 mM DTT, 1 mM EDTA), 1.2 µg calf-thymus DNA and 3 µl 20% Ficoll 400 solution. Binding reactions were kept on ice for 15 minutes and 5-8 µl was loaded onto a 5% non-denaturing polyacrylamide gel. Dried gels were exposed onto storage phosphor screen and scanned by Storm 840 PhosphorImager.

DNase I protection footprinting experiments were performed according to (2). ^{32}P single-end-labelled fragments were incubated in scaled-up (50 µl) binding reactions containing different amount of AcaCD protein. Before DNaseI treatment, 5 µl portions of the reactions were loaded onto a 5% non-denaturing polyacrylamide gel to monitor binding. The rest of the samples was treated with 5 µl of DNaseI solution (0.06 units/µl) freshly diluted from stock solution in 1x binding buffer supplemented with 10 mM $MgCl_2$ and 10 mM $CaCl_2$. Cleavage was allowed to proceed for 30 seconds at room temperature then 150 µl stop solution (20 mM Tris-HCl pH 8.0, 50 mM EDTA, 0.7% SDS and 3 µg/ml tRNA) was added. Samples were subjected to ethanol precipitation after phenol-chloroform extraction. DNaseI cleavage products were analyzed on 6% sequencing gel.

Text S3. Primer extension analysis

Total RNA was isolated from TG90 strain containing the test plasmids pJKI995 (P_{int}) or pJKI1003 (P_{xis}) w/o *AcaCD*-producer plasmid pJKI888, respectively. Overnight cultures were diluted 1000× and grown to an OD_{600} of 0.6 in 10 ml LB without antibiotics at 37 °C (cultures containing pJKI1003 were supplemented with 0.5 mM IPTG to induce transcription of the activator), then cells were harvested from 2.5 ml cultures and frozen in liquid nitrogen. To the frozen pellet 750 µl of lysis buffer (0.2 M Na-acetate pH 5.2, 1% SDS 10 mM EDTA) was added, after vortexing the mix was boiled for 2-3 min, then intensively vortexed again for 2 min. Lysates were centrifuged (20 min, 16000 rcf, 4 ºC) and the supernatants were extracted with 750 µl phenol, and centrifuged again (10 min, 16000 rcf, 4 ºC). Extraction was repeated with 720 µl phenol:chloroform (1:1), and with 360 µl chloroform. RNA was precipitated by adding 200 µl 10M LiCl to 600 µl supernatant followed by incubation on ice (1 hour) and centrifugation (10 min, 16000 rcf, 4 ºC). Pellet was first washed with 2.5 M LiCl, then with 70% ethanol, centrifuged, air dried and dissolved in 50 µl RNase-free water at 50 ºC. 20 µl (~10 µg) of total RNA was digested with 50 units of RNase-free DNaseI (Qiagen) in a final volume of 50 µl (10 min, 37 ºC), then the enzyme was inactivated (10 min, 65 ºC). The reaction mix was phenol-chloroform extracted, washed twice with 70% ethanol and dissolved in 20 µl RNase-free water after drying. RNA concentration was set to 0.5 µg/µl using NanoDrop1000 Spectrophotometer (Thermo Scientific). To control the quality 1 µg of each RNA sample was run on a 1% TAE agarose gel. Primer extension assay was performed using RevertAid H Minus first strand cDNA synthesis kit (Fermentas). One µl of pUCfor21 primer (10 µM) was labelled in 10 µl volume with 10 units of polynucleotide kinase (Fermentas) using 50 µCi [γ-^{32}P] dATP (45 min, 37 ºC) followed by inactivation of the enzyme (10 min, 68 ºC). Approximately 5 µg of RNA and 2 pmol of ^{32}P-labeled primer were mixed, heated to 70 °C for 5 min, and then allowed to anneal at 37 °C for 5 min. Extension reactions were carried out in RT buffer (50 mM Tris-HCl [pH 8.3], 50mM KCl, 4 mM $MgCl_2$ 10 mM DTT) with 1 mM dNTP and 20 u RiboLock ribonuclease inhibitor in a total volume of 20 µl (42 ºC, 60 min) using 200 u of reverse transcriptase. Extension products were precipitated by ethanol, resuspended in 3 µl DEPC-treated water, and combined with 2 µl sequencing loading solution. The sequence ladder for plasmids pJKI995 and pJKI1003 was generated with primer pUCfor21 using a Sequenase version 2.0 DNA sequencing kit (USB) according to the manufacturer's protocol. The products of each reaction were electrophoresed on a 6% denaturing polyacrylamide gel at 1800V. The gel was exposed to storage phosphor screen and scanned on Storm 840 PhosphorImager (Amersham Biosciences).

Text S4. One-step gene KO experiments

Since R55 has multiple resistance genes (*floR, cat, aadB, oxa21, qacEΔ1, sulI, mer*), this plasmid is not applicable for gene KO, thus we used its close relative, R16a for deletion of *acaCD* genes. This $Km^RAp^RSu^R$ IncA/C plasmid is self-transferable and also capable of SGI1 mobilization (3). The KO fragment was amplified from pKD3 template using primers delflhDCfor – delflhDCrev.

For construction of mini SGI1 in TG1Nal::SGI1-C, primers sgi1minidelfor – sgi1minidelrev were used to amplify the KO fragment. Distal region of P_{xis} w/o the AcaCD-protected region was deleted by using KO fragments amplified from pKD3 template with primers sgiPxisdelfor2 – sgiPxisdelrev and sgiPxisdelfor1 – sgiPxisdelrev, respectively. Orfs S*006*-*S007* were deleted using primers sgi006delfor – sgi007delrev.

The ~2.2 kb region of pJKI665 containing the *oriT*$_{RK2}$-Cm^R cassette, which was inserted into the 3' end of orf *S026* and flanked by ca. 550 bp regions of *S025* and *S026*, was amplified using primers sgi1S025rev and sgi1S026for. The amplicon was used for a one-step recombination (4) into SGI1-C in strains ST19S1, ST21S1 and ST28S1 (5), respectively. Recombinase producer plasmid pKD46 was cured at 42 ºC and the structure of Cm^R recombinants and the lack of template plasmid pJKI665 were verified by PCRs.

Text S5. Plasmid constructs

To construct the PCR template plasmid pJKI665, the 150 bp *oriT* region of RK2 plasmid (positions 51115-51276 bp) was amplified from pRK2013 (6) using primers mobfor – mobrev and the amplicon was inserted into the SalI site of pJKI309 (7), resulting in pJKI664. The same amplicon was also inserted into the SalI site of pJKI88 (7) to construct pJKI806 control plasmid. The 24321-25453 bp region of SGI1 (all SGI1 coordinates are given according to the sequence GenBank: AF261825.2) containing orfs *S025-S026* partially was amplified and cloned into BamHI-SalI site of pJKI88, resulting in pJKI653. The $oriT_{RK2}$-Cm^R cassette from pJKI664 was then inserted into the unique XbaI site of pJKI653 located near the 3' end of orf *S026*, resulting in pJKI665.

The p15A-based Km^R cloning vector pJKI691, which allows blue-white selection, was constructed for library preparation from pJKI88. First, the original MCS was replaced with a single NotI site by cloning the annealed NotIlinkerEP1 and 2 to EcoRI-PstI site (pJKI687). The unique HindIII site in the Km^R gene of pJKI687 was eliminated by imperfect filling with Klenow polymerase resulting in the Km^R vector pJKI688. Finally, the 633 bp NotI fragment of pBeloBac11 (GenBank: U51113) containing *lac*Zα gene with a MCS was inserted into the NotI site of pJKI688, leading to pJKI691.

pJKI813 carrying the 167510-354 bp BglII fragment of R55 (GenBank: JQ010984) was isolated from the BamHI-BglII library as the shortest clone (1D2) capable of activating SGI1 excision. Plasmids pJKI814 and pJKI815/pJKI816 were generated by subcloning of the 2.7 kb BstYI and the 1.62 kb SacI fragments of pJKI813 into BamHI and SacI sites of pJKI691, respectively. Homologs of pP99-019_orf133 and orf134 were inactivated in pJKI816 by insertion of $Sm^R\Omega$ fragment from pHP45Ω into StuI site in orf134 and by filling in the MfeI site in orf133, resulting in pJKI820 and pJKI824, respectively. To obtain the full length putative operon of *acaCD* genes the 2410 bp segment of R55 containing the upstream region and orfs identical to P99018_orf131, orf132 and partially the orf133 was amplified using primers 1D2for – 1D2rev and inserted into the EcoRI-StuI site of pJKI815. The resulting plasmid pJKI828 contains the 166138-169131 bp segment of R55. Plasmids pJKI829 and pJKI830 were generated by filling in the BseJI and BglII sites in pJKI828, respectively, by Klenow polymerase leading to inactivation of the first two orfs (homologs of P99018_orf131 and orf132) of the operon. Gm^R gene amplified from pJQ200SK (8) with primers GmRforSX and GmRrevSX was inserted as SalI fragment into XhoI site of pJKI828 inactivating the Km^R gene and resulting in pJKI839.

For construction of a p15A-based expression vector, the XhoI site in the Km^R gene of pJKI88 was eliminated without affecting the Km^R phenotype by imperfect filling in with Klenow polymerase. Subsequently, the 4-193 bp region of pKK223-3 (9) containing P_{tac} was amplified using primers pkkfor – BXhNdtac and cloned into EcoRI-BamHI site and then *lac*I^q from pDMI,1 (10) was inserted into the SalI site resulting in pJKI391. *AcaCD* genes were amplified from R55 (167704-168860 bp) using primers flhDNdefor – flhCBamrev and the 1156 bp amplicon was cloned into NdeI-BamHI site of pJKI391 yielding pJKI888. The same amplicon was also cloned into NdeI-BamHI site of pET22b+ resulting in pJKI878. In order to purify the regulator on chitin column, the two orfs were amplified from R55 as a single amplicon using flhDNdefor – flhCSaprev primers and cloned into SmaI site of pBluescript SK vector generating pJKI918. The insert was then cloned into NdeI-SapI site of pTXB1 expression vector (NEB) leading to pJKI958, which expressed native $FlhD_{IncA}$ and $FlhC_{IncA}$ C-terminally fused to intein-chitin binding domain (intein-CBD).

For β-galactosidase assays a pBR322-based plasmid was constructed. The EcoRI and ClaI sites of pBR322 were eliminated by recircularization of the large blunt-ended EcoRI-ClaI vector fragment (pJKI848), then the AlwNI-SspI fragment of pJKI848 containing the Ap^R gene was replaced with the similar fragment of pBluescript-SK to eliminate PstI and HincII sites (pJKI850). The promoterless *lac*Z gene cassette was inserted into HindIII-SalI site of pJKI850 as a 3.3 kb HindIII-XhoI fragment from pCEP4-LZ (J.P. Herman, unpublished), which eliminated the Tc^R marker (pJKI855). To insert MCS and supplement the first 25 bp and SD-box of *lac*Z missing from pCEP4-LZ, the chromosomal *lac*Z fragment from *E. coli* w3350 was amplified using primers mcs-laczfor – lacZ-Clarev and cloned into pJKI855 with HindIII-ClaI (pJKI860).

For transcriptional isolation of *lacZ* from outside promoters, the $Sm^R\Omega$ fragment of pHP45Ω and the *rrnB* terminator from pKK223-3 were inserted upstream of *lacZ* into pJKI860. The $Sm^R\Omega$ fragment was inserted into the unique HindIII site resulting in pJKI861. The 4123-4471 bp fragment of pKK223-3 containing the *rrnB* terminator was amplified using primers rrnBfor – rrnBXbarev2 and inserted into SmaI site of pBluescript-SK (pJKI988). The BamHI-PstI fragment from pJKI988 was cloned into BglII-PstI site of pJKI861 leading to the final construct pJKI990.

The upstream region of SGI1 *int* gene (P_{int}: 143-369 bp) and the shortened promoter region (P_{int} short: 269-364 bp) were amplified using primer pairs sgi_promIntfor – sgi_promIntrev and sgi_promIntfor2 – sgi_promIntrev and inserted into PstI-NcoI site of pJKI861 resulting in pJKI863 and pJKI870, respectively. The *rrnB* terminator was inserted into BglII-PstI site of pJKI863 from pJKI988 resulting in pJKI995. The upstream region of SGI1 *xis* gene (P_{xis}: 1947-2306 bp) was amplified with primers sgi_promXisfor – sgi_promXisrev and cloned into PstI-NcoI site of pJKI990 leading to pJKI1003. After filling in the NcoI sticky end, the PstI-NcoI fragment of pJKI1003 was cloned into PstI-SmaI site of pEMBL19 (11) leading to pPP701. The NcoI-HindIII fragment of pPP701 containing P_{xis} was isolated after filling in the NcoI sticky end and cut into two fragments with DraI. The proximal fragment (filled NcoI-DraI, 1947-2100 bp) was inserted into SmaI site of pEMBL19 resulting in pPP703 (insert is in the same direction as *lacZ*), while the distal fragment (DraI-HindIII, 2101-2306 bp) was cloned into HincII-HindIII site of pEMBL19 leading to pPP704. Additional β-gal tester plasmids were constructed as follows. The proximal P_{xis} fragment from pPP703 was cloned into pJKI990 with XbaI-NcoI resulting in pJKI991. The distal fragment from pPP704 was cloned into pJKI990 with EcoRI-PstI generating pJKI992. The BfmI-NcoI fragment of pPP701 (1947-2078 bp) was cloned into XbaI-NcoI site of pJKI990 after filling in the BfmI and XbaI sticky ends, resulting in pJKI1004. The 1947-2113 bp region of P_{xis} was amplified using primers sgi_prom*Xis*for – sgi_prom*Xis*rev2, the amplicon was cut with NcoI and inserted into NcoI-filled XbaI site of pJKI990 leading to pJKI1005. For further shortening the proximal P_{xis} fragment, the primers sgi_promXisrev3-sgi_promXisrev6 were used with sgi_promXisfor to amplify 1947-2052/2043/2030/2012 bp segments, and the amplicons were cloned into PstI-NcoI site of pJKI990, resulting in plasmids pJKI1013 – pJKI1016, respectively. Plasmid pJKI1017, which is analogous to pJKI1005, but lacks the putative -35 box nearest to TSS, was constructed by cloning the NcoI-digested amplicon obtained with primers sgi_promXis35mutlfor – sgi_promXisrev2 into SmaI-NcoI site of pJKI990. The 432 bp upstream region of *S007* was amplified with primers S007promfor – S007promrev and the amplicon was cloned into the NcoI-PstI site of pJKI990 resulting in pMSZ945.

To construct vectors expressing $FlhDC_{SGI1}$ from orfs *S007-S006*, the 7628-6481 bp segment of SGI1 was amplified using primers S007_Ncfor – S006_Bamrev and the amplicon was cloned after BamHI cleavage into SmaI-BamHI site of pBluescript SK providing pGMY2. The insert was cloned into the NcoI-BamHI site of pET16b+ resulting in pGMY3.
For cloning purposes Pwo polymerase (Roche) was applied and the cloned PCR products were sequenced. Southern hybridization was performed using Hybond-N nylon membrane (Amersham). DNA blots and labelled DNA probe were made using the DIG DNA Labelling and Detection Kit (Boehringer Mannheim) according to the manufacturer's instructions.

Text S6. Identification of R55 genes responsible for SGI1 excision and segregation

For library preparation, R55 was conjugated from TG1Nal/R55 into the plasmid-free Sm^R *E. coli* strain HB101. Plasmid DNA was isolated from 500 ml O/N LB+Cm culture using Qiagen Plasmid Maxi Kit and dissolved in 60 µl EB buffer. Ten µl DNA was digested with enzymes BamHI-BglII and EcoRI-MfeI (both combinations cut ca. 40-50 sites in R55) and ligated into BamHI or EcoRI site of pJKI691. Host strain for R55 libraries was created by transferring SGI1-C into TG1Nal from strain ST21S1/R55 (5). Transconjugants were selected on LB+Nal+Sm+Sp plates and after selection of clones lacking R55, SGI1-C integration into attB site was verified by PCRs specific for DRL, DRR_{Ec} and attP to exclude concatemeric SGI1 integrants (12). Ligated libraries were electroporated into TG1Nal::SGI1-C strain and plated on LB+Km+X-gal+IPTG. After O/N incubation at 37 °C, white colonies from each library were transferred into six 96-well plates containing 300 µl

LB+Km+Sm+10% glycerol and grown O/N at 37 ºC. From each well of every row of the plates 25 µl of O/N culture was removed and pooled (48 pools/library). 150 µl of pooled cultures was grown in 2 ml LB+Km to stationary phase and the remaining libraries in the 96-well plates were kept at -70 ºC. Total DNA was prepared from the 48 pools of both libraries and dissolved in 200 µl TE. To test the coverage of our libraries, pooled cultures were dropped onto LB+Cm, LB+Gm and LB+Ap plates. BamHI-BglII library yielded 5 Gm^R, 4 Ap^R and 20 Cm^R pools, while 10 Gm^R, and 12 Ap^R pools were found in EcoRI-MfeI library (no Cm^R pools were obtained here as EcoRI and MfeI cut in *cat* and Flo^R genes, respectively) indicating the multiple coverage of both libraries.

Screening of the pooled samples of 576 clones was carried out by attP specific PCR using 20× diluted total DNA templates and primers LJ2 and RJ4. Five $attP^+$ clones were identified in the BamHI-BglII library (Fig. S3A), while none was found in the EcoRI-MfeI library. Plasmid DNAs from the $attP^+$ clones were isolated and transformed into TG1Nal::SGI1-C strain to check their ability to generate attP signal. The verified plasmids were analysed by restriction mapping. The ca. 3.6 kb EcoRI-PstI fragment from pJKI813 (Fig. S3B) was applied as probe in Southern hybridization to examine whether the other $attP^+$ clones shared a common region. The end sequencing of insert in pJKI813 was performed with primers pUCfor21 and pUCrev22.

Since the complete sequence of R55 was not available at the time of the experiments, and later, when R55 sequence (GenBank: JQ010984) was published (13), it turned out that the annotation for this region is different from that of the previously described IncA/C family members (Fig. S3C), the 3.6 kb insert in pJKI813 was identified by using the sequence and nomenclature of close relative IncA/C plasmid pP99-018 (GenBank: AB277723), which showed 100% identity to our sequence. The 3.6 kb BglII fragment from R55 corresponded to the 114116-117770 bp segment of pP99-018 containing five intact orfs (homologs of P99018_orf133-orf137). Orf133 and orf134 appeared to be the last two genes of a four-gene putative operon, while orfs135-137 encoded on the complementary strand probably constituted a three-gene operon.

SUPPLEMENTARY REFERENCES

1. Maxam,A.M. and Gilbert,W. (1977) A new method for sequencing DNA. Proc. Natl. Acad. Sci. U. S. A., 74, 560–564.
2. Galas,D.J. and Schmitz,A. (1978) DNAse footprinting: a simple method for the detection of protein-DNA binding specificity. Nucleic Acids Res., 5, 3157–3170.
3. Douard,G., Praud,K., Cloeckaert,A. and Doublet,B. (2010) The Salmonella genomic island 1 is specifically mobilized in trans by the IncA/C multidrug resistance plasmid family. PLoS One, 5, e15302.
4. Datsenko,K.A. and Wanner,B.L. (2000) One-step inactivation of chromosomal genes in Escherichia coli K-12 using PCR products. Proc. Natl. Acad. Sci. U. S. A., 97, 6640–6645.
5. Kiss,J., Nagy,B. and Olasz,F. (2012) Stability, entrapment and variant formation of Salmonella genomic island 1. PLoS One, 7, e32497.
6. Figurski,D.H. and Helinski,D.R. (1979) Replication of an origin-containing derivative of plasmid RK2 dependent on a plasmid function provided in trans. Proc. Natl. Acad. Sci. U. S. A., 76, 1648–1652.
7. Kiss,J. and Olasz,F. (1999) Formation and transposition of the covalently closed IS30 circle : the relation between tandem dimers and monomeric circles. Mol. Microbiol., 34, 37–52.
8. Quandt,J. and Hynes,M.F. (1993) Versatile suicide vectors which allow direct selection for gene replacement in gram-negative bacteria. Gene, 127, 15–21.
9. Brosius,J. and Holy,A. (1984) Regulation of ribosomal RNA promoters with a synthetic lac operator. Proc. Natl. Acad. Sci. U. S. A., 81, 6929–6933.
10. Certa,U., Bannwarth,W., Stüber,D., Gentz,R., Lanzer,M., Le Grice,S., Guillot,F., Wendler,I., Hunsmann,G. and Bujard,H. (1986) Subregions of a conserved part of the HIV gp41 transmembrane protein are differentially recognized by antibodies of infected individuals. EMBO J., 5, 3051–3056.
11. Dente,L., Cesareni,G. and Cortese,R. (1983) pEMBL: A new family of single stranded plasmids. Nucleic Acids Res., 11, 1645–1655.
12. Doublet,B., Golding,G.R., Mulvey,M.R. and Cloeckaert,A. (2008) Secondary chromosomal attachment site and tandem integration of the mobilizable Salmonella genomic island 1. PLoS One, 3, e2060.
13. Doublet,B., Boyd,D., Douard,G., Praud,K., Cloeckaert,A. and Mulvey,M.R. (2012) Complete nucleotide sequence of the multidrug resistance IncA/C plasmid pR55 from Klebsiella pneumoniae isolated in 1969. J. Antimicrob. Chemother., 67, 2354–2360.

Figure S1

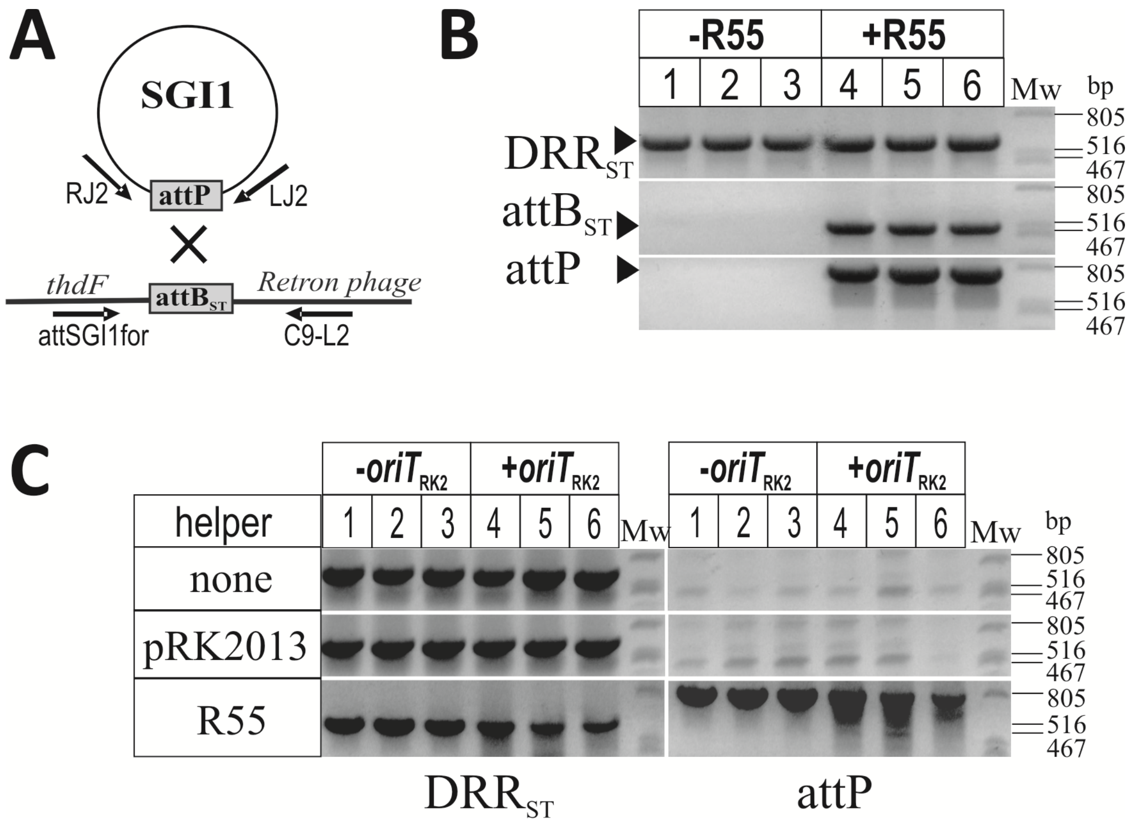

Figure S1. The IncA/C plasmid R55 encodes a specific factor that promotes SGI1 excision. (A) Schematic representation of excised SGI1. Primers used for detection of attP, $attB_{ST}$, DRL and DRR_{ST} are shown. C9-L2, which anneals to the retron phage sequence, is specific for *S.* Typhimurium and it was applied to discriminate DRR and attB of *S.* Typhimurium and *E. coli*. (B) Induction of SGI1 excision by R55 in *S.* Typhimurium strains. Chromosomally integrated SGI1 was detected in genomic DNA by PCR specific for DRR_{ST} (RJ2 – C9-L2). Excision was monitored in the same samples by PCRs specific for $attB_{ST}$ (attsgi1for – C9-L2) and attP (LJ2 – RJ2). Lanes 1-3: wt ST1369, ST1375 and ST1773 without R55; lanes 4-6: ST1369, ST1375 and ST1773 derivatives carrying R55. Mw, molecular weight ladder – λ DNA digested with PstI. (C) Induction of SGI1 excision by R55 and pRK2013 helper plasmid in *S.* Typhimurium strains harbouring SGI1-C:: $oriT_{RK2}$. Lanes 1-3: wt ST19S1, ST21S1 and ST28S1; lanes 4-6: ST19S1, ST21S1 and ST28S1 carrying $oriT_{RK2}$ integrated into SGI1-C. First row: no helper plasmid, second row: pRK2013 helper plasmid, third row: R55 helper plasmid. Colony PCRs detecting DRR_{ST} and attP were carried out with primers shown in panel B.

Figure S2

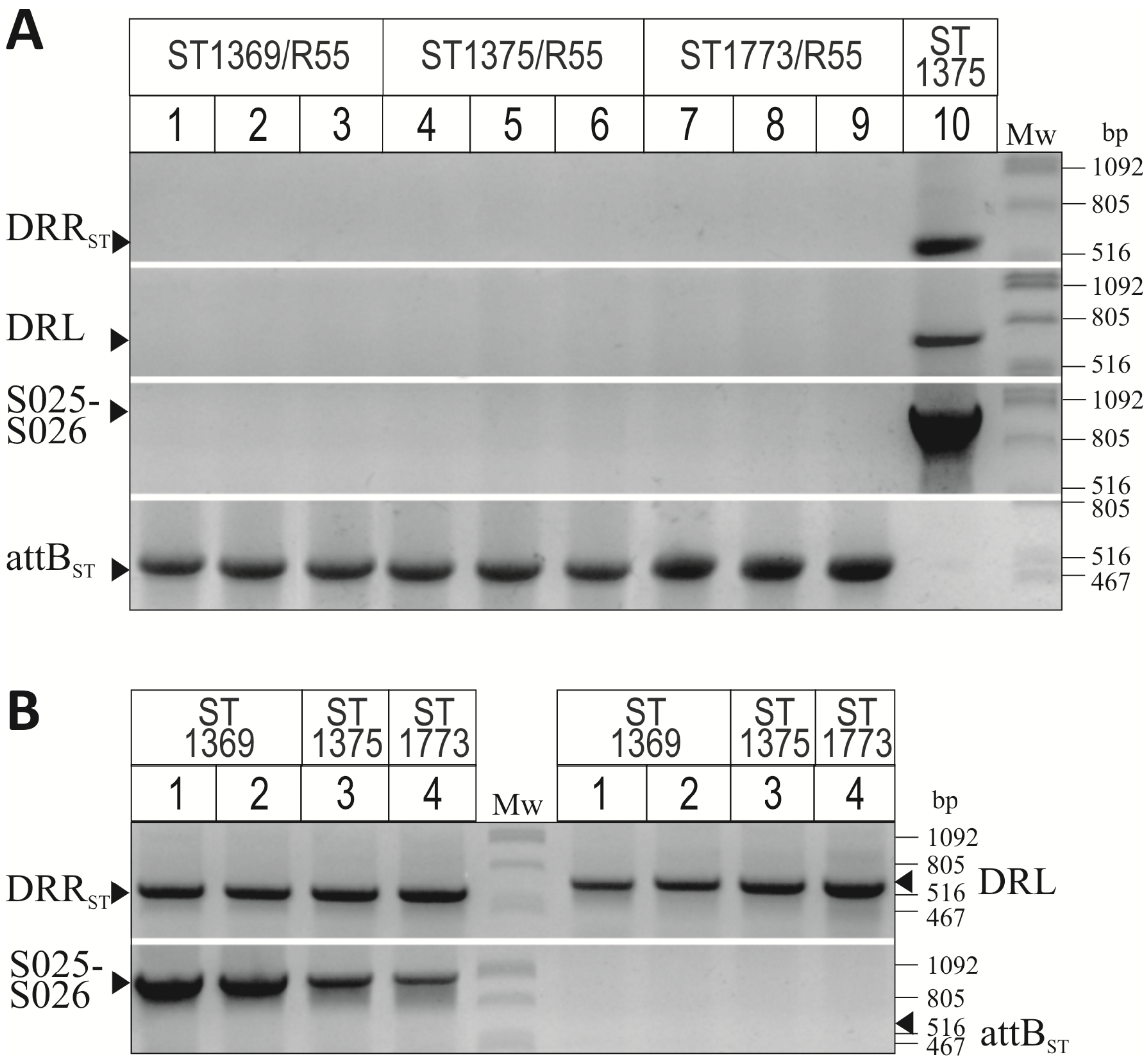

Figure S2. Verification of the presence or absence of SGI1 in Tc^S colonies obtained from the first and tenth passages of three *S.* Typhimurium strains. The strains ST1369, ST1375, ST1773 and their derivatives containing R55 were passaged without selection for SGI1. Colony PCRs were carried out with the following primers: DRR_{ST}, RJ2 – C9-L2; $attB_{ST}$, attsgi1for – C9-L2; attP, LJ2 – RJ2; inner segment of SGI1, sgi1S025rev – sgi1S026for. (A) Lanes 1-9: colony PCRs of three randomly selected Tc^S clones from the first passage of the three *Salmonella* strains harbouring R55. Lane 10: colony PCRs of the Tc^S clone isolated from the first passage of ST1375 showing that SGI1 is present in the original position. (B) Colony PCRs for the 4 Tc^S clones isolated from the tenth passage of the three strains lacking R55 (Table 1).

Figure S3

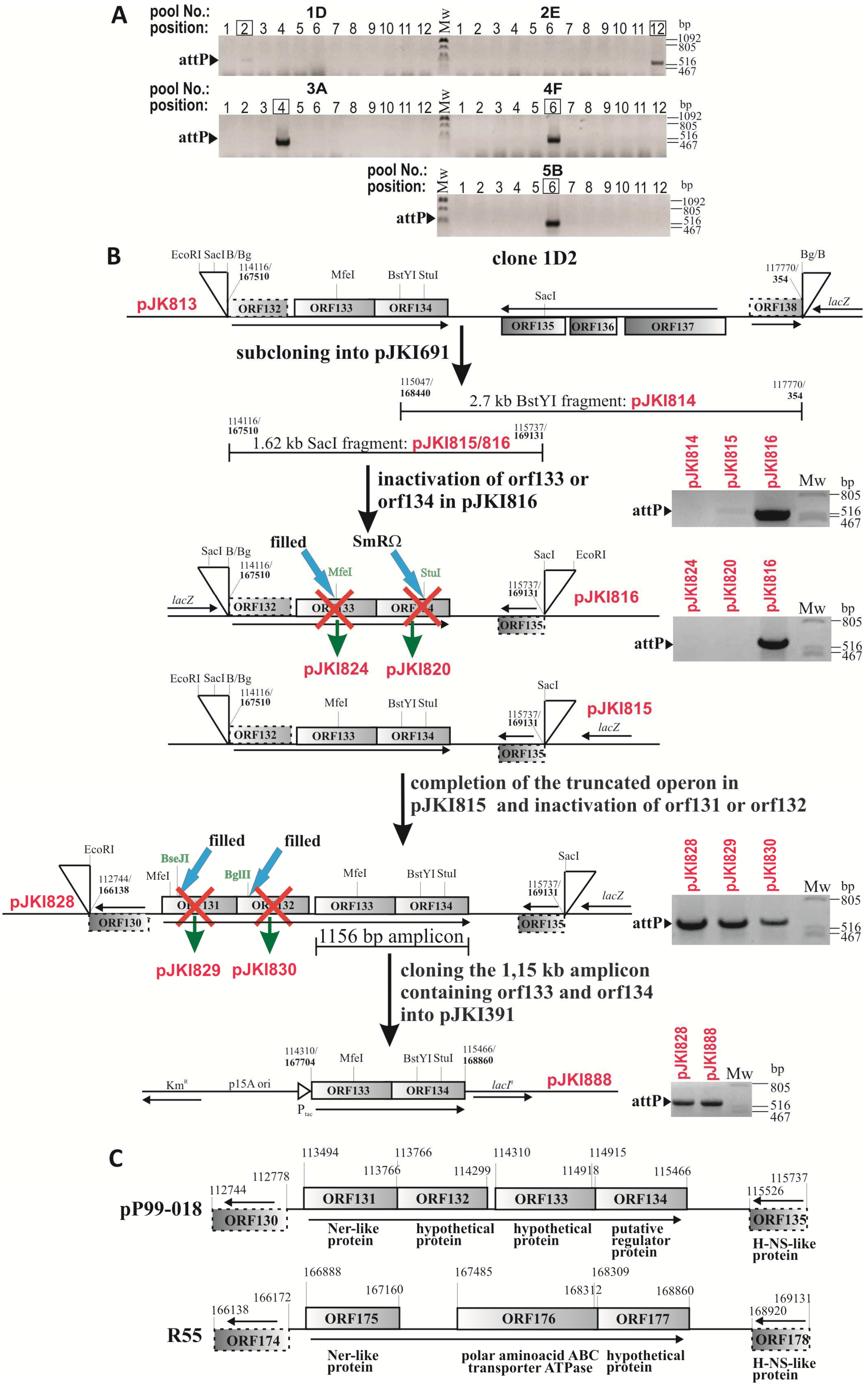

A
pool No.:
position:
1D
2E
3A
4F
5B
attP
Mw
bp
1092
805
516
467
B
clone 1D2
pJK813
EcoRI SacI B/Bg
Bg/B
MfeI
BstYI StuI
SacI
ORF132
ORF133
ORF134
ORF135
ORF136
ORF137
ORF138
lacZ
subcloning into pJKI691
2.7 kb BstYI fragment: pJKI814
1.62 kb SacI fragment: pJKI815/816
inactivation of orf133 or orf134 in pJKI816
filled
SmRΩ
pJKI816
pJKI824
pJKI820
pJKI815
pJKI814
completion of the truncated operon in pJKI815 and inactivation of orf131 or orf132
pJKI828
BseJI
BglII
ORF130
1156 bp amplicon
pJKI829
pJKI830
cloning the 1,15 kb amplicon containing orf133 and orf134 into pJKI391
pJKI888
p15A ori
C
pP99-018
ORF131
Ner-like protein
hypothetical protein
putative regulator protein
H-NS-like protein
R55
ORF174
ORF175
ORF176
ORF177
ORF178
polar aminoacid ABC transporter ATPase

Figure S3. Identification of gene(s) responsible for SGI1 excision. (A) Detection of attP$^+$ clones in the BamHI-BglII library (Text S6). The twelve individual colonies represented in each attP$^+$ pool (1D, 2E, 3A, 4F and 5B) were tested by attP specific colony PCR (primers LJ2 – RJ4). AttP$^+$ clones are indicated. (B) Identification of gene(s) promoting SGI1 excision on plasmid pJKI813 from 1D2 clone. pJKI813 contains the 3654 bp BglII fragment of R55. The 3.6 kb fragment corresponds to the 114116-117770 bp segment of pP99-018 containing orf132-138. Numbering of orfs is given according to pP99-018 annotation since annotation of R55 for this region is different from that of the previously described IncA/C family members (Text S6). Corresponding coordinates of pPP99-018 and R55 are shown as normal/bold, respectively. First, the two operon-like segments encoded on the complementary strands were subcloned. Plasmid pJKI814 was obtained by cloning the 2.7 kb BstYI fragment containing three intact genes, orf135-137, while pJKI815 and pJKI816 were generated by cloning the 1.62 kb SacI fragment in opposite directions. The two intact orfs (orf133-134) were expressed from P_{lac} in pJKI816, while in pJKI815 there was no true promoter to drive these genes. AttP specific colony PCRs of TG1Nal::SGI1-C colonies transformed with pJKI814, pJKI815 and pJKI816 showed that SGI1-C excision was promoted exclusively by orf133-134 in an expression-level-dependent manner (compare the signals of the promoterless pJKI815 and pJKI816 expressing orf133-134 from P_{lac}). The two orfs were then separately inactivated in pJKI816 by filling in the MfeI site in orf133 (pJKI824) or inserting the $Sm^R\Omega$ cassette into StuI site in orf134 (pJKI820). AttP test for pJKI824 and pJKI820 transformant TG1Nal::SGI1-C colonies was negative, indicating that both orfs are necessary for SGI1 excision. To obtain the complete putative operon of the *acaCD* genes, the 2410 bp segment containing the upstream region and orf131-134 was amplified from R55 and inserted into the EcoRI-StuI site of pJKI815, resulting in pJKI828. Orf131 and orf132 were independently inactivated in pJKI828 by filling in the BseJI (pJKI829) and BglII (pJKI830) sites, respectively. AttP tests for pJKI828, pJKI829 and pJKI830 transformant TG1Nal::SGI1-C colonies showed that the complete operon and both KO derivatives activated SGI1 excision. Finally, orf133-134 was amplified and placed under the control of P_{tac} in pJKI888, which also caused strong attP signal in PCR. Dashed line boxes indicate orfs truncated via cloning, triangles represent multicloning sites (only relevant restriction sites are shown), B/Bg indicates hybrid sites generated by ligation of BamHI and BglII compatible ends. (C) Different annotations of the same sequence in pP99-018 and R55. Annotated orfs and their start and end positions are given according to the published sequences GenBank: AB277723 and JQ010984, respectively.

Figure S4

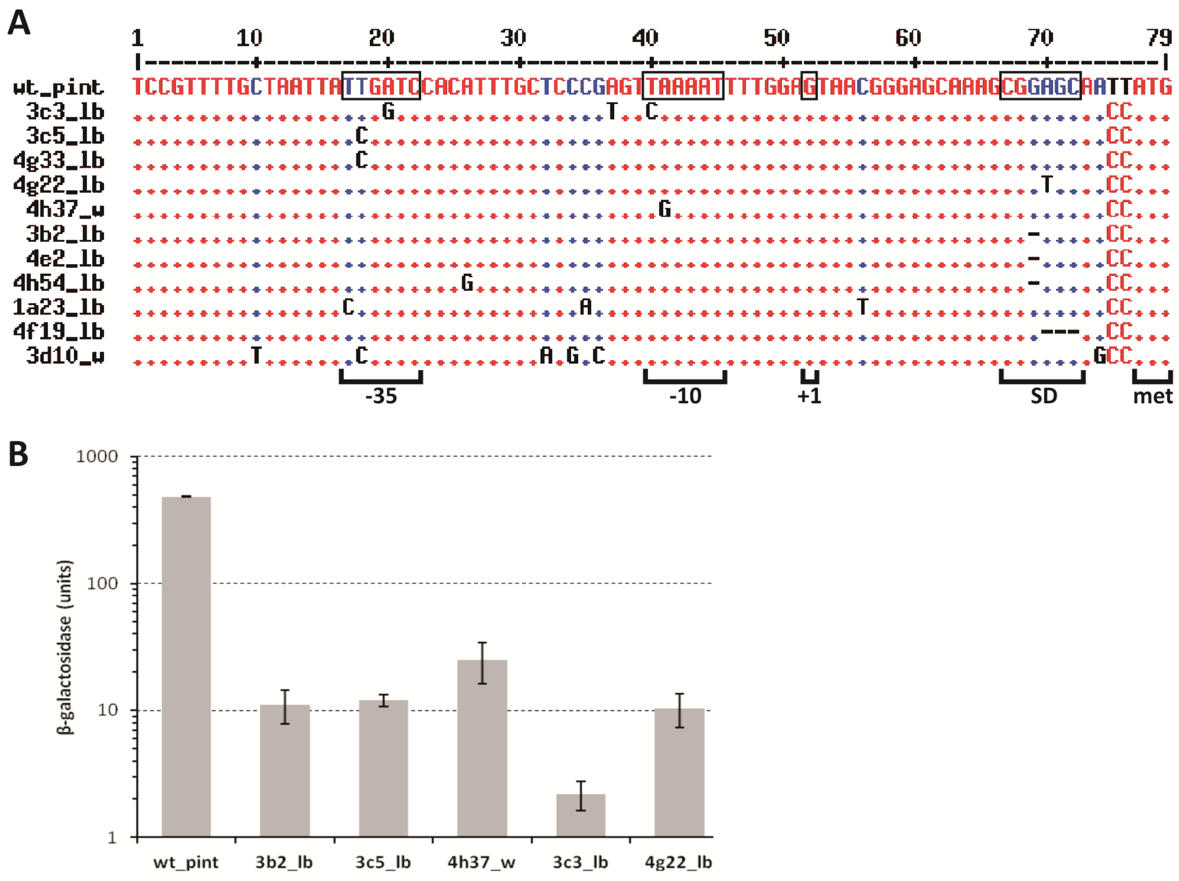

Figure S4. Analysis of P_{int} promoter mutants. (A) Alignment of P_{int}_short sequences obtained from the random PCR mutagenesis experiment. First two characters in the clone designations refer to the PCR protocol resulting the mutant (Text S1), while ‘lb’ and ‘w’ indicate the colour of the mutant (light blue or white) in β-gal drop test. The potential Shine-Dalgarno (SD), -10, -35 elements are boxed and indicated below the alignment together with TSS and start codon of *int*. Bases differing from the wt sequence are indicated in the alignment. Note that the two positions preceding the ATG (start codon of *lacZ*) had to be changed to generate an NcoI site for cloning the amplicons into the β-gal tester plasmid pJKI861. The alignment was generated using the MultAlin interface. (B) β-galactosidase assay for representative promoter mutants.

Figure S5.

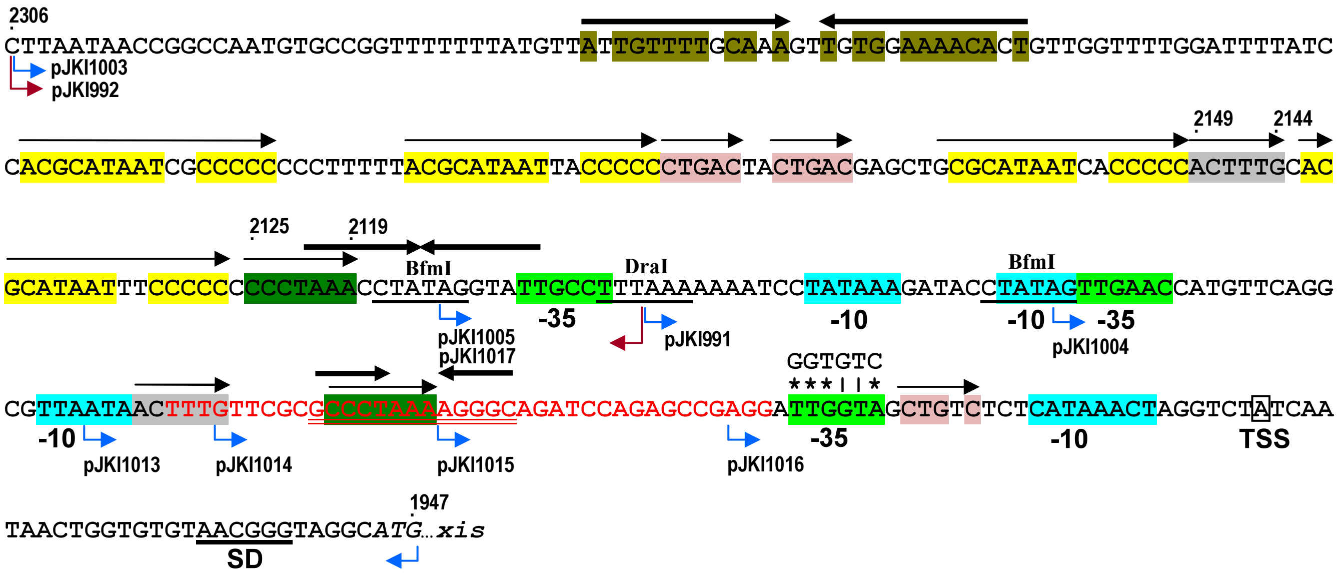

Figure S5. Schematic representation of P_{xis} region. The sequence carries different directly and inversely repeated elements that are colour-coded and shown above the sequence by thin and thick arrows, respectively. The sense strand is shown in 5'→3' direction from 2306 to 1947 bp (the non-coding region between *xis* and orf S003). The putative -35 and -10 boxes are highlighted by green and blue, respectively, ATG of *xis* is indicated by italics. The endpoints of P_{xis} fragments cloned into β-gal tester plasmids are shown below the sequence by coloured arrows: red, pJKI992; blue, all the other constructs that share the ATG codon of *xis* as endpoint (the corresponding plasmid names are indicated near the 5' end of the fragment). In pJKI1017 the putative -35 box was replaced for 'ggtgtc' sequence, mismatches are indicated by asterisks. Potential SD sequence and TSS of *xis* mRNA are indicated. Restriction sites applied in cloning are underlined and indicated above the sequence. The region protected by AcaCD is shown in red letters. The 13 bp IR element (core AcaCD binding site) is double underlined. The 7 bp CCCTAAA motif in the IR element and the 6 bp ACTTTG motif overlapping the 5' end of protected area are repeated in direct orientation in the distal part of P_{xis} region (2125-2119 bp and 2149-2144bp, respectively).

Figure S6

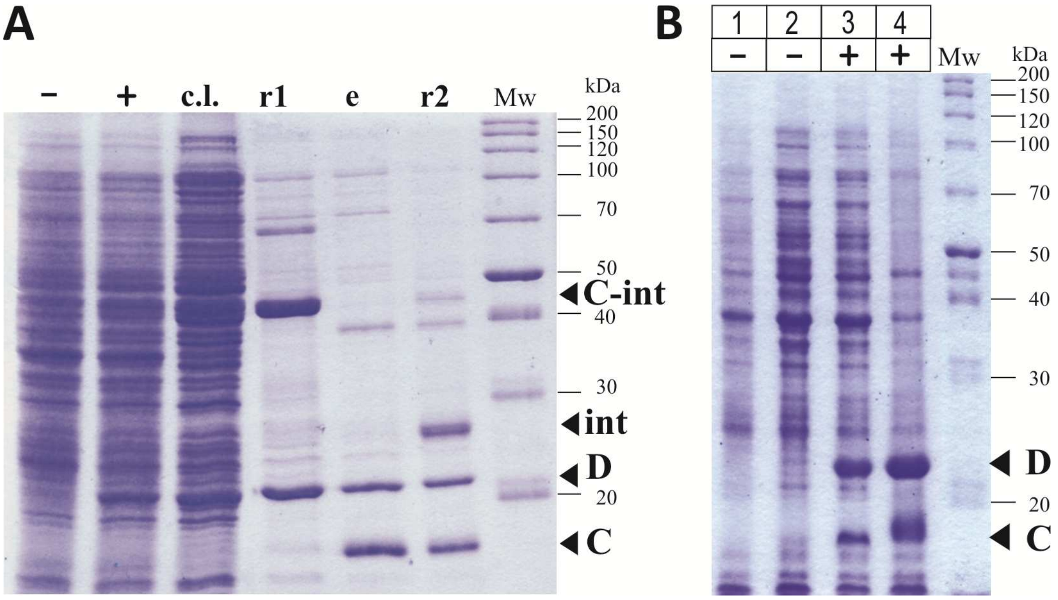

Figure S6. (A) Purification of AcaCD (Text S2). The native D subunit and the C subunit fused to the *Mxe* intein-CBD was expressed in *E. coli* Tuner (DE3) from pJKI958 containing the two orfs of *acaCD* under the control of P_{T7}. The SDS-PAGE analysis showed that AcaCD binds to the tethered AcaC-intein-CBD (lane r1) and they co-elute after induction of self-cleavage by the intein tag (lane e). Large amount of AcaD protein remains bound to the resin after cleavage suggesting that the complex tends to denature when present in high concentration (r2). Lanes '–' and '+': total protein preparation from non-induced and induced Tuner/pJKI958 cultures. Protein samples: c.l., cleared lysate; r1, washed resin before cleavage; e, eluted protein after self-cleavage by intein; r2, total protein extracted from the chitin resin after elution; Mw, protein ladder. The AcaD (D), AcaC-intein-CBD fusion protein (C-int), free AcaC (C) and intein-CBD (int) protein bands are indicated. (B) Expression of $FlhDC_{SGI1}$ from orfs *S007-S006*. The SDS-PAGE shows that $FlhDC_{SGI1}$ can be overexpressed from P_{T7} promoter in *E. coli* Tuner (DE3). Lane 1, negative control (pET16b); lanes 2-3, orfs *S007-S006* under the control of P_{T7} (pGMY3); lane 4, *acaCD* genes under the control of P_{T7} (pJKI878). Symbols '–' and '+' refer to non-inducing and inducing (0 or 0.5 mM IPTG for 2h at 37 ºC) culture conditions applied before total protein preparation. FlhD and FlhC of SGI1 and subunits of AcaCD from R55 are indicated as D and C, respectively.

Figure S7

A

	AcaC	SetC$_{SXT}$	FlhC$_{ST}$	FlhC$_{Ec}$
FlhC$_{SGI1}$ (S006)	**79/89**[a]	**33/49**	**28/43**	**27/43**
	AcaD	SetD$_{SXT}$	FlhD$_{ST}$	FlhD$_{Ec}$
FlhD$_{SGI1}$ (S007)	**46/67**	**21/45**	**25/44**	**29/50**

[a] Identity/similarity (%)

B

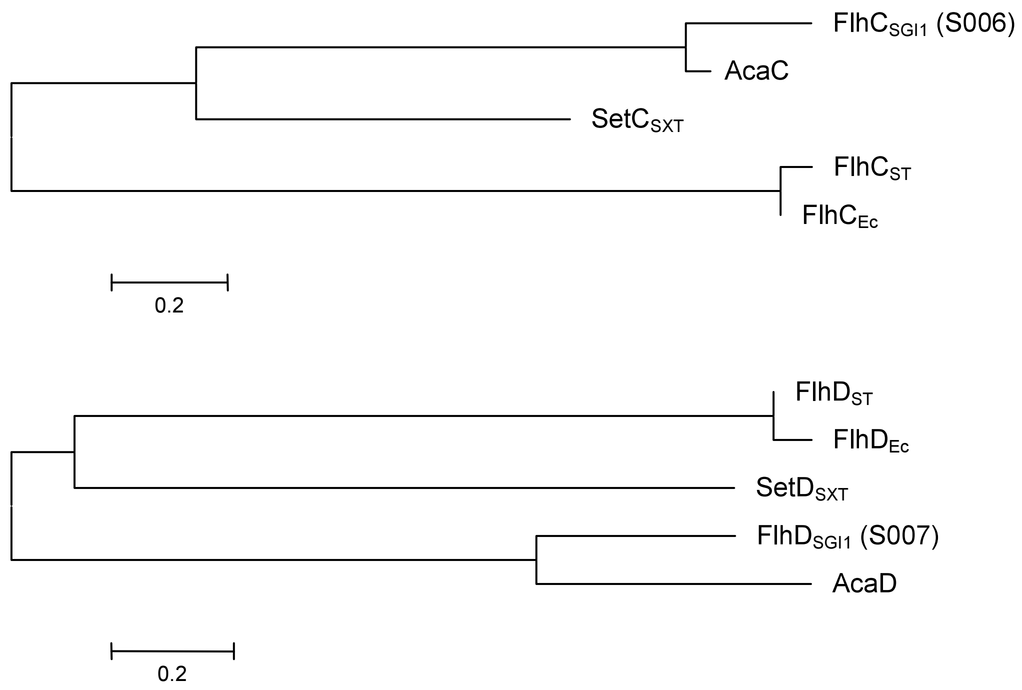

Figure S7. Sequence based comparison of FlhDC$_{SGI1}$ protein to FlhDC family regulators AcaCD, SetCD of SXT and FlhDC from *E. coli* and *Salmonella*. (A) Identities and similarities between the protein sequences. Data were generated by pair-wise alignment using protein Blast. (B) Phylogenetic reconstruction for the FlhD and FlhC proteins by Maximum likelihood method.

Table S1. Oligonucleotide primers used.

Name	Sequence (5'→3')	References
sgi1S025rev	aaggatcctcgtctacggtgttacttttcaac	this work
sgi1S026for	aagtcgacctctttatacaatcgtcctag	this work
attsgi1for	gctctagagcggccgcatggaaggcggcttcctggc	(1)
attsgi1rev	gctctagagcggccgcaaatgtgaatcgaatcacaatcg	(1)
C9-L2	agcaagtgtgcgtaatttgg	(2)
LJ2	agctgcagcggccgcaagttttactctgtcttccag	(1)
RJ2	agctgcagcggccgctcgaagaggtagagcag	(1)
RJ4	ctttatgtgttttgtgattggtaag	this work
sgi1seqfor	gagatcatctgcaggtgactgtaatc	(1)
sgi1seqrev	aactgcagtggttgggtatacttcagc	(1)
mobfor	tacgtcgacggtagccggccagcctcg	this work
mobrev	cgtgtcgactttccttggtgtatcc	this work
NotIlinkerEP1	aattgcggccgcttgca	this work
NotIlinkerEP2	agcggccgc	this work
pUCfor21	cagggttttcccagtcacgac	(3)
pUCrev22	tcacacaggaaacagctatgac	(3)
1D2for	cagaattcccagcagaagaatattgaccac	this work
1D2rev	cattagaagagaggcctgtatc	this work
BXhNdtac	cgggatccctcgagcatatgtgtttcctgtgtgaaattgt	this work
flhDNdefor	ggccatatgttggaactggatattattgg	this work
flhCBamrev	ataggatcctcaagcacactctttctccccac	this work
flhCSaprev	ggtggttgctcttccgcaagcacactctttctccccac	this work
GmRforSX	aagtcgactctagattgacataagcctgttcggttc	this work
GmRrevSX	aagtcgactctagattaggtggcggtacttgggtc	this work
delflhDCfor	gagtcttggctatctatcgcaaccttcgtgatttgtgagggggggcggaatGTGTAGGCTGGAGCTGCTTC	this work
delflhDCrev	cgcaaagtttgggcgctcatcttctggtccgaaatgtcatagtctactCATATGAATATCCTCCTTAGTTC	this work
sgi1minidelfor	ctttgcaaaacaataacataaaaaaaccggcacattggccggttattaagGTGTAGGCTGGAGCTGCTTC	this work
sgi1minidelrev	acttaccaatcacaaaacacataaagtattgattatatgaagatattactCATATGAATATCCTCCTTAGTTC	this work
sgiPxisdelfor1	caccagttattgatagacctagtttatgagagacagctaccaatcctCATATGAATATCCTCCTTAGTTC	this work
sgiPxisdelfor2	caatcctcggctctggatctgcccttttagggcgcgaacaaagttatCATATGAATATCCTCCTTAGTTC	this work
sgiPxisdelrev	gcggaattctgatgatgtccagcccccaccgattccataccgtaattgaGTGTAGGCTGGAGCTGCTTC	this work
sgi006delfor	aattagcatcgctatttgcccttttgcggcatacgcggatgtattcaCATATGAATATCCTCCTTAGTTC	this work
sgi007delrev	tagtttcaatcaatctgggccgcagaaaaaaaggtaaggaggactactgaGTGTAGGCTGGAGCTGCTTC	this work
S006_Bamrev	ttggatcctcaggcagcttttgagacccggc	this work
S007_Ncfor	ttccatggatgccttagagccgttagaacg	this work
mcs-lacZfor	gggaagcttaggcctagatctctgcagtctagagtcgacgcggccgcggatcccccgggaattcaagaaggagatataccatg gccatgattacggattcactggc	this work
lacZ-Clarev	aaccaccacgctcatcgataatttc	this work
rrnBfor	aactcgaggatcctggcggcagtagcgcggtgg	this work
rrnBrev2	aatctagagtcgacaaacaaaagagtttgtagaaacgc	this work
sgi_promIntfor	aactgcagcttctgtattgggaagtaaatctcc	this work
sgi_promIntfor2	aactgcagacttcccttctcacgattttgc	this work
sgi_promIntrev	cctccatggttgctccgctttgctcccgttac	this work
sgi_promXisfor	cagccatgggcctacccgttacacaccag	this work
sgi_promXisrev	aactgcagcttaataaccggccaatgtgccgg	this work
sgi_promXisrev2	aggtattgcctttaaaaaaatcctataaag	this work
sgi_promXisrev3	ttctgcagataactttgttcgcgccctaaaag	this work
sgi_promXisrev4	ttctgcagttcgcgccctaaaagggcagatc	this work
sgi_promXisrev5	ttctgcagggcagatccagagccgagga	this work
sgi_promXisrev6	ttctgcaggattggtagctgtctctcataaactag	this work
sgi_promXis35mutlfor	cagccatgggcctacccgttacacaccagttattgatagacctagtttatgagagacagcgacacctcctcggctctgg	this work
S007promfor	atccatgggtagtcctccttaccttttttctgc	this work
S007promrev	ttctgcagcgattaaaaacaaagagaatagcgccttttttc	this work

Restriction sites are underlined, uppercase indicate the 3' part of KO oligos annealing to the template plasmid pKD3, positions of base changes in sgi_promXis35mutlfor oligo are double underlined.

SUPPLEMENTARY REFERENCES

1. Kiss,J., Nagy,B. and Olasz,F. (2012) Stability, entrapment and variant formation of Salmonella genomic island 1. PLoS One, 7, e32497.
2. Boyd,D.A., Peters,G.A., Ng,L. and Mulvey,M.R. (2000) Partial characterization of a genomic island associated with the multidrug resistance region of Salmonella enterica Typhymurium. FEMS Microbiol. Lett., 189, 285–291.
3. Kiss,J. and Olasz,F. (1999) Formation and transposition of the covalently closed IS30 circle : the relation between tandem dimers and monomeric circles. Mol. Microbiol., 34, 37–52.

Table S2. Bacterial strains used in this study

Strains	Genotype or relevant features[a]	References
E. coli		
TG1	*supE hsd*Δ*5 thi*Δ(*lac-proAB*) F'[*traD36 proAB*$^+$ *lacI*^{q} *lacZΔM15*]	(1)
TG1Nal	Nal^R derivative of TG1	(2)
TG1Nal::SGI1-C	Nal^{R}Sm^{R}Sp^{R}Su^R, SGI1-C transconjugant of TG1Nal. Single copy of SGI1-C is integrated in attB (*trmE*).	This work
TG1Nal::miniSGI	Nal^{R}Cm^{R}Sm^{S}Sp^{S}Su^S deletion derivative of TG1Nal::SGI1-C (2307-42469 bp region is replaced by Cm^R gene)	This work
TG1Nal::SGI1-C/R16a	TG1Nal::SGI1-C containing R16a, Nal^{R}Sm^{R}Sp^{R}Su^{R}Km^{R}Ap^R	This work
TG1Nal/R55	TG1Nal containing R55, Nal^R,Ap^R,Cm^R,Flo^R,Su^R,Km^R,Gm^R	This work
HB101	F$^-$ *supE*44 *hsdS20*(r_B^- m_B^-) *recA13 ara-14 proA2 thi lacY1 galK*2 *leu rpsL20 xyl*-5 *mtl-1 glnV44*	(3)
HB101/R55	HB101 containing R55, Ap^R,Cm^R,Flo^R,Sm^R,Su^R, Km^R,Gm^R	This work
TG2	*supE hsd*Δ*5 thi*Δ(*lac-proAB*)Δ(*srl-recA*)306::Tn*10*(Tc^R) F'[*traD36 proAB*$^+$ *lacI*^{q} *lacZΔM15*]	(4)
TG90	*pcn B80 zad*::Tn*10* (Tc^R) derivative of TG1	(5)
TG90Nal	Nal^R derivative of TG90	(2)
S17-1	*pro thi recA hsdR* (r^- m^+) Tp^R Sm^R,Sp^R Km^S[Ω RP4-2-Tc::Mu-Km::Tn*7*], competent in mobilization of *oriT*$_{RK2}$	(6)
S17-1Nal	Nal^R S17-1 derivative	This work
S17-1Nal::SGI1-C:: *oriT*$_{RK2}$	Nal^{R}Sm^{R}Sp^{R}Su^{R}Cm^R, SGI1-C::*oriT*$_{RK2}$ transconjugant of S17-1Nal. Single copy of SGI1 is integrated in attB (*trmE*).	This work
Tuner (DE3)	F$^-$ *ompT hsdS$_B$*(r_B^- m_B^-) *gal dcm lacY1* (DE3)	Novagen
Tuner::SGI1-C	Sm^{R}Sp^{R}Su^R, SGI1-C transconjugant of Tuner(DE3). Single copy of SGI1-C is integrated in attB (*trmE*).	This work
S. e. Typhimurium.		
LT2 MA1703	*recA1*, *srl*	L. Bossi, unpublished
ST1369	wt SGI1, Ap^R,Cm^R,Flo^R,Sm^R,Sp^R,Su^R,Tc^R,Rif^R,Km^S,Gm^S,Nal^S	(7)
ST1375	wt SGI1, Ap^R,Cm^R,Flo^R,Sm^R,Sp^R,Su^R,Tc^R,Rif^R,Km^S,Gm^S,Nal^S	(7)
ST1773	wt SGI1, Ap^R,Cm^R,Flo^R,Sm^R,Sp^R,Su^R,Tc^R,Rif^R,Km^S,Gm^S,Nal^S	(7)
ST19S1	SGI1-C, Ap^S,Cm^S,Flo^S,Sm^R,Sp^R,Su^R,Tc^S,Rif^R,Km^S,Gm^S,Nal^S derivative of *S.* T. 1369	(2)
ST21S1	SGI1-C, Ap^S,Cm^S,Flo^S,Sm^R,Sp^R,Su^R,Tc^S,Rif^R,Km^S,Gm^S,Nal^S derivative of *S.* T. 1375	(2)
ST28S1	SGI1-C, Ap^S,Cm^S,Flo^S,Sm^R,Sp^R,Su^R,Tc^S,Rif^R,Km^S,Gm^S,Nal^S derivative of *S.* T. 1773	(2)
ST19S1::*oriT*$_{RK2}$	SGI1-C::*oriT*$_{RK2}$-Cm^R, Ap^S,Cm^R,Flo^S,Sm^R,Sp^R,Su^R,Tc^S,Rif^R,Km^S,Gm^S,Nal^S derivative of *S*T19S1	This work
ST21S1::*oriT*$_{RK2}$	SGI1-C::*oriT*$_{RK22}$-Cm^R, Ap^S,Cm^R,Flo^S,Sm^R,Sp^R,Su^R,Tc^S,Rif^R,Km^S,Gm^S,Nal^S derivative of *S*T21S1	This work
ST28S1::*oriT*$_{RK2}$	SGI1-C::*oriT*$_{RK2}$-Cm^R, Ap^S,Cm^R,Flo^S,Sm^R,Sp^R,Su^R,Tc^S,Rif^R,Km^S,Gm^S,Nal^S derivative of *S*T28S1	This work
ST1369/R55	wt SGI1, R55, Ap^R,Cm^R,Flo^R,Sm^R,Sp^R,Su^R,Tc^R,Rif^R,Km^R,Gm^R,Nal^S	(2
ST1375/R55	wt SGI1, R55, Ap^R,Cm^R,Flo^R,Sm^R,Sp^R,Su^R,Tc^R,Rif^R,Km^R,Gm^R,Nal^S	(2)
ST1773/R55	wt SGI1, R55, Ap^R,Cm^R,Flo^R,Sm^R,Sp^R,Su^R,Tc^R,Rif^R,Km^R,Gm^R,Nal^S	(2)
ST19S1/R55	SGI1-C, R55, Ap^R,Cm^R,Flo^R,Sm^R,Sp^R,Su^R,Tc^S,Rif^R,Km^R,Gm^R,Nal^S	(2)
ST21S1/R55	SGI1-C, R55, Ap^R,Cm^R,Flo^R,Sm^R,Sp^R,Su^R,Tc^S,Rif^R,Km^R,Gm^R,Nal^S	(2)
ST28S1/R55	SGI1-C, R55, Ap^R,Cm^R,Flo^R,Sm^R,Sp^R,Su^R,Tc^S,Rif^R,Km^R,Gm^R,Nal^S	(2)
ST19S1::*oriT*$_{RK2}$/R55	SGI1-C::*oriT*$_{RK2}$-Cm^R, R55, Ap^R,Cm^R,Flo^R,Sm^R,Sp^R,Su^R,Tc^S,Rif^R,Km^R,Gm^R,Nal^S	This work
ST21S1::*oriT*$_{RK2}$/R55	SGI1-C::*oriT*$_{RK2}$-Cm^R, R55, Ap^R,Cm^R,Flo^R,Sm^R,Sp^R,Su^R,Tc^S,Rif^R,Km^R,Gm^R,Nal^S	This work
ST28S1::*oriT*$_{RK2}$/R55	SGI1-C::*oriT*$_{RK2}$-Cm^R, R55, Ap^R,Cm^R,Flo^R,Sm^R,Sp^R,Su^R,Tc^S,Rif^R,Km^R,Gm^R,Nal^S	This work

[a] Ap, ampicillin; Cm, chloramphenicol;Flo, florfenicol; Gm, gentamicin; Km, kanamycin; Nal, nalidixic acid; Rif, rifampicin; Sm, streptomycin; Sp, spectinomycin; Su, sulphonamides; Tc, tetracycline; $^{R/S}$, resistant/sensitive

SUPPLEMENTARY REFERENCES

1. Gibson,T.J. (1984) Studies on the Epstein-Barr virus genome. Thesis.
2. Kiss,J., Nagy,B. and Olasz,F. (2012) Stability, entrapment and variant formation of Salmonella genomic island 1. PLoS One, 7, e32497.
3. Boyer,H.W. and Roulland-Dussoix,D. (1969) A complementation analysis of the restriction and modification of DNA in Escherichia coli. J. Mol. Biol., 41, 459–472.
4. Sambrook,J., Fritsch,E.F. and Maniatis,T. (1989) Molecular Cloning: A Laboratory Manual. Cold Spring Harbor Laboratory Press, Cold Spring Harbor, NY.
5. Gonzy-Treboul,G., Karmazyn-Campelli,C. and Stragier,P. (1992) Developmental regulation of transcription of the Bacillus subtilis ftsAZ operon. J. Mol. Biol., 224, 967–979.

6. Simon,R., Priefer,U. and Pühler,A. (1983) A Broad Host Range Mobilization System for In Vivo Genetic Engineering: Transposon Mutagenesis in Gram Negative Bacteria. Bio/Technology, 1, 784–791.
7. Fekete,P.Z. and Nagy,B. (2008) Salmonella Genomic Island 1 (SGI1) and genetic characteristics of animal and food isolates of Salmonella Typhimurium DT104 in Hungary. Acta Vet. Hung., 56, 5–11.

Table S3. Relevant features of plasmids used in this study.

Name	Relevant features[a]	References
pJKI88	Km^R, p15A cloning vector	(1)
pJKI309	Ap^R, Cm^R, pMB1. pEMBL19 derivative harbouring *cat* gene	(1)
pJKI391	pJKI88 derivative expression vector containing P_{tac} followed by a MCS and $lacI^q$ gene	this work
pJK648	Ap^S, Gm^R derivative of pKD46, Gm^R cassette is inserted into the Ap^R gene	this work
pJKI664	pJKI309 derivative containing the $oriT_{RK2}$ inserted next to the *cat* gene	this work
pJKI653	pJKI88 derivative containing the 24321-25453 bp region (parts of *S025-S026* orfs) of SGI1	this work
pJKI665	pJKI653 derivative PCR template plasmid containing $oriT_{RK2}$-Cm^R cassette flanked by ~550 bp fragments of *S025* and *S026*	this work
pJKI687	Km^R pJKI88 derivative, MCS is replaced by a single NotI site	this work
pJKI688	Km^R pJKI687 derivative, HindIII site is eliminated in the Km^R gene	this work
pJKI691	pJKI88 derivative cloning vector containing MCS-*lacZ*α from pBeloBac11	this work
pJKI806	pJKI88 derivative containing the $oriT_{RK2}$	this work
pJKI813	pJKI691 derivative containing the 167510-(170810/1)-354 bp segment of R55	this work
pJKI814	pJKI691 derivative containing the 168440-(170810/1)-354 bp segment of R55	this work
pJKI815	pJKI691 derivative containing the 168440-169131 bp segment of R55, orfs are expressed from a weak unknown promoter	this work
pJKI816	pJKI691 derivative containing the 168440-169131 bp segment of R55, orfs are expressed from P_{lac}	this work
pJKI820	pJKI816 derivative, $Sm^R\Omega$ cassette inserted into StuI site (KO of P99018_orf134 homolog)	this work
pJKI824	pJKI816 derivative, MfeI site filled in (KO of P99018_orf133 homolog)	this work
pJKI828	pJKI815 derivative containing the 166138-169131 bp segment of R55 (the whole putative operon homologous to P99018 orf131-134)	this work
pJKI829	pJKI828 derivative, BseJI site filled in (KO of P99018_orf131 homolog)	this work
pJKI830	pJKI828 derivative, BglII site filled in (KO of P99018_orf132 homolog)	this work
pJKI839	Km^S, Gm^R derivative of pJKI828	this work
pJKI888	pJKI391 derivative containg the *acaCD* genes (167704-168860 bp) of R55 (P99018_orf133-134 homologs) under the control of P_{tac}	this work
pJKI878	pET22b derivative containing *acaCD* genes under the control of P_{T7}	this work
pJKI958	pTXB1 (NEB) derivative containing the *acaCD* genes, *acaC* is fused to *Mxe* intein-chitin binding domain gene at its 3' end.	this work
pJKI861	Basic cloning vector for β-gal assays containing promoterless *lacZ*, preceded by MCS and terminators of $Sm^R\Omega$ cassette	this work
pJKI988	pEMBL19 derivative carrying 4123-4471 bp *rrnB* terminator fragment of pKK223-3	this work
pJKI863	pJKI861 derivative tester plasmid containing the whole P_{int} region (143-369 bp of SGI1)	this work
pJKI870	pJKI861 derivative tester plasmid containing 5' shortened P_{int} region (269-369 bp of SGI1)	this work
pJKI990	pJKI861 derivative cloning vector for β-gal assays supplemented with the *rrnB* terminator	this work
pJKI991	pJKI990 derivative tester plasmid containing proximal P_{xis} fragment (1947-2100 bp of SGI1)	this work
pJKI992	pJKI990 derivative tester plasmid containing distal P_{xis} fragment (2101-2306 bp of SGI1)	this work
pJKI995	pJKI863 derivative tester plasmid containing P_{int} region (143-369 bp) preceded by *rrnB* terminator	this work
pJKI1003	pJKI990 derivative tester plasmid containing P_{xis} region (1947-2306 bp of SGI1)	this work
pJKI1004	pJKI990 derivative tester plasmid containing proximal P_{xis} fragment (1947-2078 bp)	this work
pJKI1005	pJKI990 derivative tester plasmid containing proximal P_{xis} fragment (1947-2113 bp)	this work
pJKI1013	pJKI990 derivative tester plasmid containing proximal P_{xis} fragment (1947-2052 bp)	this work
pJKI1014	pJKI990 derivative tester plasmid containing proximal P_{xis} fragment (1947-2043 bp)	this work
pJKI1015	pJKI990 derivative tester plasmid containing proximal P_{xis} fragment (1947-2030 bp)	this work
pJKI1016	pJKI990 derivative tester plasmid containing proximal P_{xis} fragment (1947-2012 bp)	this work
pJKI1017	pJKI990 derivative tester plasmid containing proximal P_{xis} fragment (1947-2113 bp) with mutated -35 box (2003-2008 bp)	this work
pGMY2	pBluescript II-SK derivative carrying orfs *S007-S006* of SGI1	this work
pGMY3	pET-16b derivative expressing orfs *S007-S006* of SGI1 from P_{T7} promoter	this work
pPP701	pEMBL19 derivative containing P_{xis} (1947-2306 bp of SGI1)	this work
pPP703	pEMBL19 derivative containing proximal P_{xis} fragment (1947-2100 bp of SGI1)	this work
pPP704	pEMBL19 derivative containing distal P_{xis} fragment (DraI-HindIII, 2101-2306 bp)	this work
R55	IncA/C tra+$Ap^RKm^RCm^RFlo^RGm^RSu^R$	(2)
R16a	IncA/C, tra+,$Ap^RKm^RSu^R$	(2)

pRK2013	Km^R, colE1, RK2 derivative with all transfer functions	(3)
pKD46	ara-inducible expression of λ Red recombinase, temperature-sensitive pSC101 replication system	(4)
pKD3	Cm^R, Ap^R r6kγ-based PCR template plasmid for one-step recombination gene-KO	(4)
pHP45ΩSm^R	Ap^R,Sm^R,Sp^R, harbouring the Sm^R/Sp^R cassette flanked by transcriptional and translational terminators	(5)
pKK223-3	ColE1-based Ap^R expression vector, P$_{tac}$	(6)
pEMBL19	pMB1 based Ap^R cloning vector	(7)
pBluescript II-SK	pMB1 based Ap^R cloning vector	(8)
pTXB1	Ap^R expression vector for *Mxe* intein-chitin binding domain tagging, P$_{T7}$	NEB
pET-16b	Ap^R expression vector , P$_{T7}$	Novagen
pET-22b	Ap^R expression vector , P$_{T7}$	Novagen
pMSZ945	pJKI990 derivative tester plasmid containing the upstream region of *S007* (7626-8057 bp of SGI1)	this work

[a] MCS, multicloning site

SUPPLEMENTARY REFERENCES

1. Kiss,J. and Olasz,F. (1999) Formation and transposition of the covalently closed IS30 circle : the relation between tandem dimers and monomeric circles. Mol. Microbiol., 34, 37–52.
2. Douard,G., Praud,K., Cloeckaert,A. and Doublet,B. (2010) The Salmonella genomic island 1 is specifically mobilized in trans by the IncA/C multidrug resistance plasmid family. PLoS One, 5, e15302.
3. Figurski,D.H. and Helinski,D.R. (1979) Replication of an origin-containing derivative of plasmid RK2 dependent on a plasmid function provided in trans. Proc. Natl. Acad. Sci. U. S. A., 76, 1648–1652.
4. Datsenko,K.A. and Wanner,B.L. (2000) One-step inactivation of chromosomal genes in Escherichia coli K-12 using PCR products. Proc. Natl. Acad. Sci. U. S. A., 97, 6640–6645.
5. Prentki,P. and Krisch,H.M. (1984) In vitro insertional mutagenesis with a selectable DNA fragment. Gene, 29, 303–313.
6. Brosius,J. and Holy,A. (1984) Regulation of ribosomal RNA promoters with a synthetic lac operator. Proc. Natl. Acad. Sci. U. S. A., 81, 6929–6933.
7. Dente,L., Cesareni,G. and Cortese,R. (1983) pEMBL: A new family of single stranded plasmids. Nucleic Acids Res., 11, 1645–1655.
8. Short,J.M., Fernandez,J.M., Sorge,J.A. and Huse,W.D. (1988) Lambda ZAP: A bacteriophage lambda expression vector with in vivo excision properties. Nucleic Acids Res., 16, 7583–7600.

Table S4. SGI1-C loss from *E. coli* TG1Nal::SGI1-C strain in the presence of plasmids expressing AcaCD activator.

No. of passage	TG1Nal::SGI1-C/ plasmid	promoter of *acaCD*	total no. of colonies	Sm^S SGI1$^-$ colonies	Rate of SGI1$^-$ colonies (%)
1	pJKI813	no	1852	2	0.2±0.1
	pJKI816	P$_{lac}$	2894	2256	77.3±2.7
	pJKI828	own	2492	722	38.8±6.9
	R55	own	4125	1576	39.8±10.1
	pJKI691[a]	-	3285	0	<0.12±0.01
5	pJKI813	no	1968	5	0.4±0.2
	pJKI816	P$_{lac}$	206	192	95.3±5.1
	pJKI828	own	380	349	90.0±7.0
	R55	own	524	232	41.6±8.9
	pJKI691[a]	-	8144	0	<0.05±0.01

[a] The cloning vector pJKI691 lacking *acaCD* served as negative control.
